# Fluorescent‐Labeled Octasilsesquioxane Nanohybrids as Potential Materials for Latent Fingerprinting Detection

**DOI:** 10.1002/chem.202001908

**Published:** 2020-09-17

**Authors:** Enock O. Dare, Victoria Vendrell‐Criado, M. Consuelo Jiménez, Raúl Pérez‐Ruiz, David Díaz Díaz

**Affiliations:** ^1^ Institute of Organic Chemistry University of Regensburg Universitaetsstr. 31 93040 Regensburg Germany; ^2^ Department of Chemistry Federal University of Agriculture P.M. B 2240 Abeokuta Nigeria; ^3^ Departamento de Química Universitat Politècnica de València Camino de Vera, s/n 46022 Valencia Spain; ^4^ Departamento de Química Orgánica Universidad de La Laguna Avda. Astrofísico Francisco Sánchez 38206 La Laguna Tenerife Spain; ^5^ Instituto Universitario de Bio-Orgánica Antonio González Universidad de La Laguna Avda. Astrofísico Francisco Sánchez 2 38206 La Laguna Tenerife Spain

**Keywords:** click chemistry, fingerprint identification, fluorophores, photostability, silsesquioxanes

## Abstract

The recent demand for fluorescent‐labeled materials (FLMs) in forensic security concepts such as latent fingerprints (LFs) that encode information for anti‐counterfeiting and encryption of confidential data makes necessary the development of building new and innovative materials. Here, novel FLMs based on polyhedral oligomeric silsesquioxanes (POSS) functionalized with fluorophores via “click” reactions have been successfully synthesized and fully characterized. A comprehensive study of their photophysical properties has displayed large Stokes's shift together with good photostability in all cases, fulfilling the fundamental requisites for any legible LF detection on various surfaces. The excellent performance of the hetero‐bifunctional FLM in the visualization of LF is emphasized by their legibility, selectivity, sensitivity and temporal photostability. In this study, development mechanisms have been proposed and the overall concept constitute a novel approach for vis‐à‐vis forensic investigations to trace an individual's identity.

## Graphical abstract

**A series of fluorescent‐labeled** nanohybrids based on polyhedral oligomeric silsesquioxanes (POSS) linked to appropriate fluorophores via “click” chemistry can be used to visualize latent fingerprints on several surfaces, providing reasonable legibility patterns that satisfy the requirements for fingerprinting identification in forensic technology.

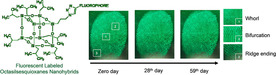

Fluorescent‐labeled materials (FLMs) are very important entities with widespread applicability, ranging from white light emitting diodes (WLED),^1^ molecular imaging,^2^ in vivo and in vitro cellular targeting and imaging,^3^ to detection of latent fingerprints (LFs) in the forensic sector.^4^, ^5^, ^6^, ^7^, ^8^, ^9^ Within this context, FLMs possess better features than classical fluorescent organic dyes,^3^, ^10^ including higher absorption coefficients and sensitivity, avoiding possible photobleaching, larger emission lifetimes and very low toxicity allowing them to be applied in vitro and in vivo analysis.^11^

Public security remains a global challenge and the utilization of LFs, bar‐codes for anti‐counterfeiting, and confidential data encryption, among others, are being currently applied by forensic institutions.^5^, ^6^, ^7^, ^8^, ^9^ In particular, LFs appears to be the best option for personnel identification in forensic investigations due to their high stability, uniqueness and complexity of ridges patterns.^9^, ^12^, ^13^ In an attempt to improve the traditional fingerprint detection methods,^14^ fluorescent nanomaterials have been found to be suitable platforms for the development of LFs.^14^ Among co‐doping of 2D nanostructures, a rigid 3D hetero‐structural scaffold could be a promising candidate due to its efficiency, photostability and effectivity.

In this regard, polyhedral oligomeric silsesquioxanes (POSS) have emerged as a valuable group of 3D nano‐building‐blocks for the fabrication of a variety of hybrid functional materials.^15^, ^16^, ^17^, ^18^, ^19^ Several prime qualifications make POSS the appropriate choice for its use as principal core: (i) it exhibits high stability and excellent biocompatibility in a biological environment,^20^ (ii) the inorganic cage provides not only protection to the covalently attached dye from stability but also a convenient framework for 3D multivalent display of pendant epitopes,^21^ (iii) facile functionalization.^22^ The highly symmetrical and topologically ideal cubic‐octameric framework (T8), with general formula (RSiO1.5)_8_ and a cage size of approximately 0.5–0.7 nm has been reported as suitable precursor.^15^ Based on literature data,^23^, ^24^, ^25^ this species can be easily functionalized through a “click” reaction without compromising its structural and functional integrity. However, as far as we are aware, examples of hybrid POSS materials bearing fluorophores as powerful tools for effective LFs detection have not been reported yet.

In this work, novel FLMs based on the 3D POSS containing fluorescent dyes have been synthesized in order to develop LF visualization materials. The synthetic strategy has followed the classical “click” reaction where the Cu^I^‐catalyzed [3+2] cycloaddition between the POSS precursor with an azide group (**PAZ**, see Scheme 1) and the corresponding alkyne‐like fluorophore has been carried out (see the SI for details). It is worth mentioning that the use of “click” chemistry has been proved to be a versatile tool for preparing a large variety of POSS conjugates for different applications,^21^, ^22^ including POSS‐dye derivatives.^26^, ^27^, ^28^, ^29^ Herein, four different materials relying on the fluorophore nature (pyrene=**PAP**; dansyl=**PAD**; bromo‐naphthalic anhydride=**PANA**; piperazine‐naphthalic=**PANP**) have been obtained (Scheme 1). Their photophysical properties as well as measurements on the photostability of these species have been investigated in detail and, finally, these FMLs have been successfully tested for the detection of LFs. The polyaromatic dyes were selected based on its commercial availability and magnificient fluorescence properties.^30^

**Scheme 1:**
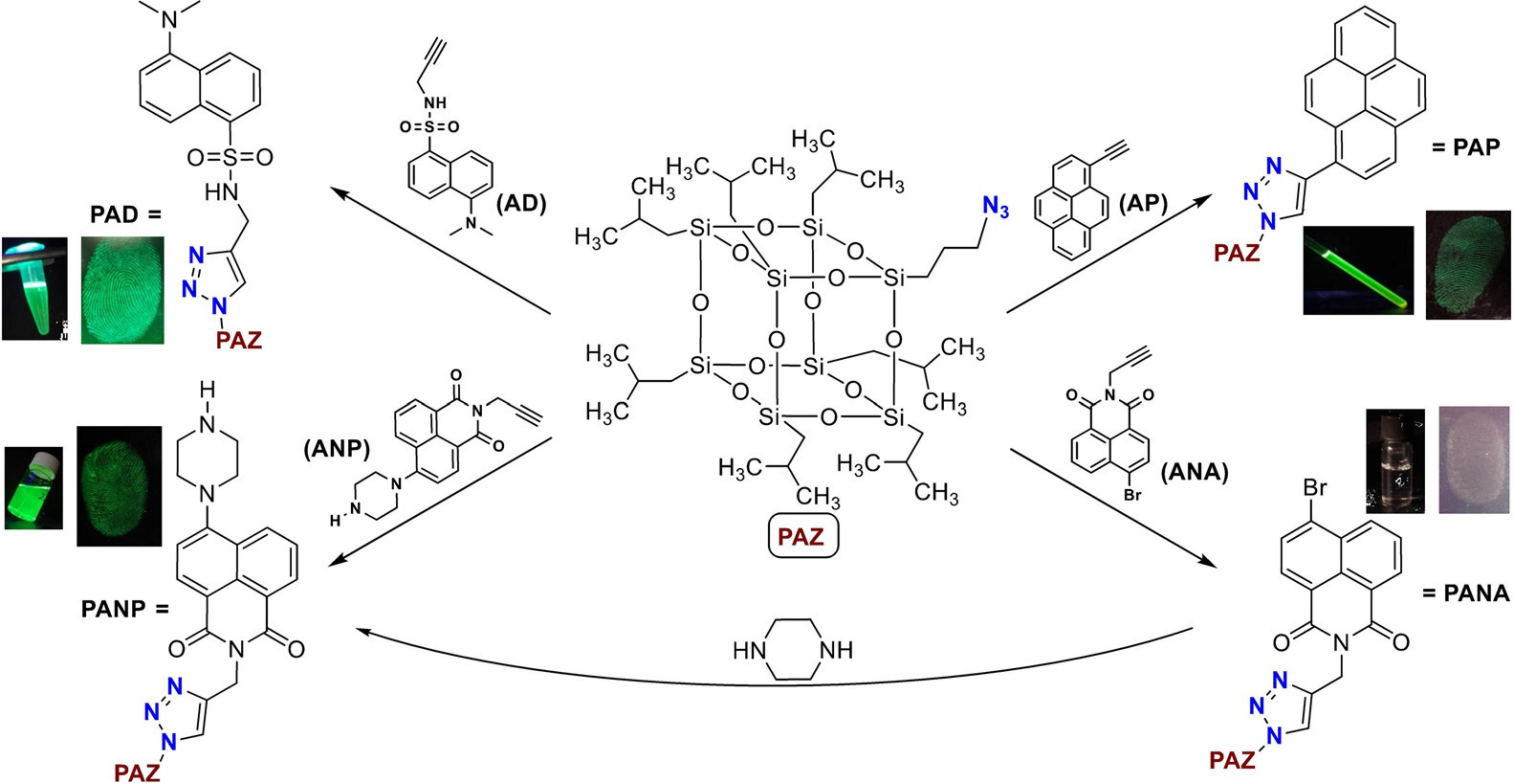
Synthetic procedure for **PAP**, **PAD**, **PANA** and **PANP**, with inset indicating their corresponding UV lamp (365 nm) irradiation and fingerprint response.

Prior to the synthesis of the corresponding FLMs, the precursors, **PAZ**,^25^ and the alkyne‐like fluorophores **AP**,^31^ **AD** 32 and **ANA** 33 were prepared following previously reported procedures (see ESI for details) where spectral data were consistent according to literature.

Next, coupling reaction between **PAZ** and the fluorophore derivatives, respectively, through a typical “click” reaction allowed the formation of **PAP**, **PAD**, **PANA** and **PANP** from good‐to‐excellent isolated yields (ca. 81 %, 74 %, 91 % and 68 %, respectively). Optimized conditions included the presence of CuBr/PMDETA as catalyst system using tetrahydrofuran (THF)/dimethylformamide (DMF) (1/1 v/v) as solvent after 18 h (Table S1). The role of PMDETA was pivotal as it scavenged intruding Cu^II^, whereas the use of THF justified the good solubility of **PAZ** in this solvent.^25^ Variation on the catalyst system, solvent mixture and reaction time did not improve the results (Table S1). Moreover, a different procedure based on direct aminolysis of **PANA** with piperazine was also attempted (Scheme 1), albeit the isolated yield was even lower (44 %) with this strategy. The molecular structures of **PAP**, **PAD**, **PANA** and **PANP** were characterized by ^1^H and ^29^Si NMR, FTIR and mass spectrometry.

The presence of the triazole proton, the methylene group in alpha position related to the triazole moiety for **PAD**, **PANA** and **PANP** and the *iso‐butyl* protons from the **PAZ** core evidenced the successful “click” reaction (Figures S1–S4). From the ^29^Si NMR spectra (Figure S5A), two peaks were detected in all cases which indicated the corresponding bi‐functionalization of the POSS material with Si atoms containing two different organic groups (Si‐*iso‐butyl* and Si‐(CH_2_)_3_‐triazole‐fluorophore). Further, these chemical shifts were in agreement not only with the molecular symmetry considerations^21^, ^23^ but also within the expected region for an alkyl‐substituted cubic POSS (ca. *δ*=−65 to −70 ppm),^21^, ^24^ supporting the T8 cage structural scaffold. All hybrid materials were analyzed by mass spectrometry. The results showed molecular ion peaks at 1126, 1188, 1215, and 1218 (*m*/*z*) for **PAP**, **PAD**, **PANA** and **PANP**, respectively, with appropriate supportive fragmentation pattern (Figure S5B). Finally, regarding FTIR measurements, Figure S6 shows a representative example of the assembling between **PAZ** and the alkyne‐like pyrene derivative where the signal of the azide group at 2050 cm^−1^ clearly disappeared together with the appearance of the typical C=C (1740 cm^−1^) of the pyrene. Indeed, the Si−O−Si bond signals (1134–1022 cm^−1^) of the T8 silsesquioxane core were also detected in the **PAP** spectrum confirming that the T8 cage was not affected by the reaction conditions or by the strong nucleophilic N_3_.^25^

Once the structural elucidation of **PAP**, **PAD**, **PANA** and **PANP** was fully detailed, we next investigated their photophysical properties in different solvents which included absorption/emission bands, molar absorption coefficient, Stoke's shifts, fluorescence quantum yields, emission rate constants and singlet energies and lifetimes (Figures S7–S11 and Tables S2–S5).

Accordingly, the alkyne‐like fluorophores were also studied in order to observe the 3D POSS core influence. To note that the presence of heavy atoms on the fluorophore moiety drops strongly the emission quantum yield as in the case of **PANA** (Table S5) and therefore this material was ruled out for further spectroscopic analysis.

Commencing with **AP** and **PAP** (Figure S7 and Tables S2A B), the UV‐vis absorption spectrum of **AP** showed three characteristic peaks in all solvents which was typically ascribed to pyrene chromophore; however, this well‐structured absorption band was not defined in the case of **PAP** where it presented only one maximum absorption.^34^, ^35^ The reason of this broad band could be only explained by the presence of the 3D POSS material anchorage to the pyrene moiety affecting the S0→S1 and S0→S2 transitions. Additionally, the most significant effect was detected on the Stoke's shifts values which were markedly higher in the case of **PAP** in comparison with **AP**. These data suggested the positive influence of the triazolyl‐POSS scaffold which promoted energy transfer between the donor and acceptor. Concerning the singlet lifetimes (Table S2B), it was clear that two components appeared at moderate‐to‐non polar solvents, indicating the contribution of two species to the emission presumably both the monomer and an excimer species (Figure S12).^36^

Regarding **AD** and **PAD** (Figure S8 and Table S3A B), remarkably differences were observed in the molar absorption coefficient (*ϵ*) where it seemed clear that *ϵ* was solvent‐dependent only in the case of **PAD**. The emission spectra in various solvents with different polarity revealed a red shift on the maximum. Thus, the emission maximum appeared at 450 nm in non‐interacting solvents (hexane, cyclohexane), whereas in moderately polar solvents such as dichloromethane (DCM) or THF the maximum was at 490–500 nm. However, the maximum red shift of the emission was provided in highly polar solvents [acetonitrile (ACN), DMF or dimethylsulfoxide (DMSO)] at ca. 520 nm where an intramolecular charge transfer (ICT) character could be facilitated.^37^ High Stoke's shift values were obtained as typical of dansylated compounds^38^ and, from the emission decay traces, a bi‐exponential fitting in all cases were achieved (Table S3B) two emission lifetimes were detected in all cases. They could be ascribed to the ICT state (shorter lifetimes) and possible aggregates (longer lifetimes). As a reference, **PAD** emission spectra show a shorter wavelength shoulder band at ≈400 nm in highly polar solvents (Figure S7).^39^ Although contribution of the longer lifetime component was found to be clearly in a minor extend, it was higher in highly polar solvents than in non‐polar solvents.^40^

Finally, **ANP** and **PANP** were also submitted to photophysical studies (Figure S9 and Tables S4A B). The most relevant difference was observed in the molar absorption coefficients, being 3‐fold higher in **PANP** and, therefore, the presence of the 3D POSS material strongly affected to the solubility of the naphthalimide derivative. Again, both **ANP** and **PANP** possessed emission bands that were found to be highly red shifted (≈54 nm and ≈54 nm, respectively) from the non‐interacting solvent hexane to polar DMF and a decrease of the fluorescence quantum yield was obtained for **PANP** especially in non‐polar solvents; this fact could be attributed to the better solubility of **PANP** that would facilitate the formation of complexes in the ground state or internal transfer processes. In this context, choice of **PANP** instead of **PANA** gave rise to a “OFF‐ON” irreversible switch system possessing “receptor‐fluorophore‐receptor” architectural model (Figure S13).^41^ Furthermore, computational calculations based on TDDFT//B3LYP/6‐31G(d) level of theory^42^ supported the use of the piperazine moiety (Table 1). Hence, the heavy atom Br present at the naphthalimide fluorophore in **PANA** induced a non‐radiative transition with oscillator strength (*f*) of 0.0028 justifying its dark state forbidden S_0_→S_1_ transition which was non‐fluorescent. The high theoretical oscillator strength (*f*=0.32) obtained for **PANP** was corroborated by a significantly high fluorescence radiative factor (*R*_fl_=5.6×10^8^) and quantum yield (*φ*_fl_=0.34). Whereas, a non‐radiative **PANA** displayed negligible zero R_fl_ and quantum yield (*φ*_fl_=0.026) in DCM.^43^ After nucleophilic substitution of Br by piperazine a radiative transition S_0_→S_1_, identified as the HOMO→LOMO was allowed as a result of electron jump within the architectural model. Therefore, the presence of emissive S1 which depends on solvents dielectric underpins the strong fluorescence of **PANP**, which is classified as polar in its excited state. Thus, the theoretical indication further supports PET mechanism. Indeed, we term this an irreversible “OFF‐ON system as there is no future reversible photo‐damaged sensing program for **PANP** as reported for most metal‐44 and thiol‐induced^45^ sensors. Indeed, terminal hydrogen of piperazine is a target for our prospective fingerprinting detection.^46^, ^47^

**Table 1:** Computational studies based on TDDFT//B3LYP/6‐31G(d) at the ground state geometry

FLM	Electronic transition	Oscillator strength (*f*)	Composition	CI
**PANA**	S_0_→ S_1_	0.0028	H → L	0.53
	S_0_→ S_4_	0.00011	H_‐1_ → L	0.09
**PANP**	S_0_→ S_1_	0.32	H → L	0.65

To check whether **PAP**, **PAD** and **PANP** were or were not stable after photolysis, absorption spectra of the corresponding materials were recorded before and after monochromatic light irradiation. As depicted in Figure 1, all these compounds exhibited a magnificent photostability even after 60 minutes of continuous irradiation. As a matter of fact, the 3D POSS scaffold linked to the corresponding fluorophores together with the triazole bridge^48^ played a crucial role for further real applications such as LFs. In this vein, similar investigations for the alkyne‐like fluorophores were carried out. In contrast to their analogous materials, they presented poor stability after prolonged exposure of light as monitored by the absorption and emission spectra (Figure S14).

**Figure 1:**
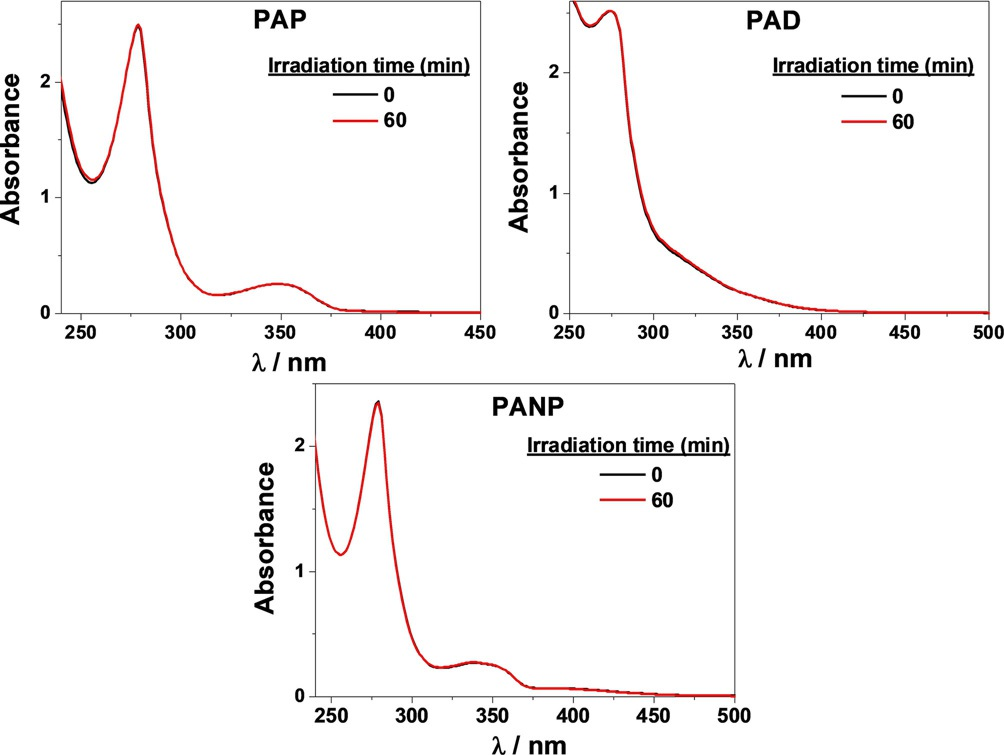
Absorption spectra of **PAP** (*λ*_exc_=340 nm), **PAD** (*λ*_exc_=340 nm) and **PANP** (*λ*_exc_=400 nm) before (black) and after (red) monochromatic light irradiation in aerated THF solution. Concentration of all samples were fixed to 0.01 mm.

Some conclusions were drawn from the photophysical data: large Stoke's shift were obtained in general, high fluorescence quantum yields were observed in some cases and an ideal photostability made these FLMs (**PAP**, **PAD** and **PANP**) potential candidates to be successfully applied as fluorescence‐based fingerprinting detection materials.

In general, many methods reported for fingerprinting detection depend on the affinity between the amino acid‐based oily components of the fingerprints and the hydrophobic compounds used in the developing reagents.^49^ In order to test the potential of our hybrid materials for this important application, we adopted two methods for fingerprints deposition: (1) direct stamping of forehead‐rubbed fingerprint on both smooth and porous substrates (e.g., glass, plastic, leather, paper), and (2) lifting up the fingerprints using the sticky side of an adhesive tape.

Figure 2 displays fresh (0 days) and aged fingerprint images (stored at RT for 28 or 59 days). The undeveloped fingerprint patterns were hardly visible regardless the illumination (UV or vis light). In contrast, those developed under diluted solution of the FLMs apparently displayed enhanced legibility due to the greater contrast between the fluorescent ridge and non‐fluorescent furrow. The brightness, contrast and visual legibility remained the same for at least three months for **PAD** and **PANP**. However, dimmer fingerprint fluorescence was observed with **PAP** after one month, probably due to loss of some components on the ridges over time. The longer temporal stability observed for **PAD** and **PANP** was ascribed to their respective structural outfit bearing functional groups capable of providing hydrogen‐bonding with the residual amino acids in the fingerprints (e.g. (**PAD**)S=O⋅⋅⋅H−N (amino acid), (**PANP**)N−H⋅⋅⋅O(amino acid)). In addition, N atoms also contribute forming strong N⋅⋅⋅H−O interactions.^14^, ^50^, ^51^, ^52^, ^53^, ^54^ Furthermore, detailed observation of the friction ridge features (see enlarged areas in Figure 2), showed whorl, bifurcation, and ridge ending, which satisfy to a large extend the requirements of fingerprint identification. Interestingly, our fingerprint development process also offered distinguished visual legibility on other surfaces, including handset phone surfaces (Figure S15).

**Figure 2:**
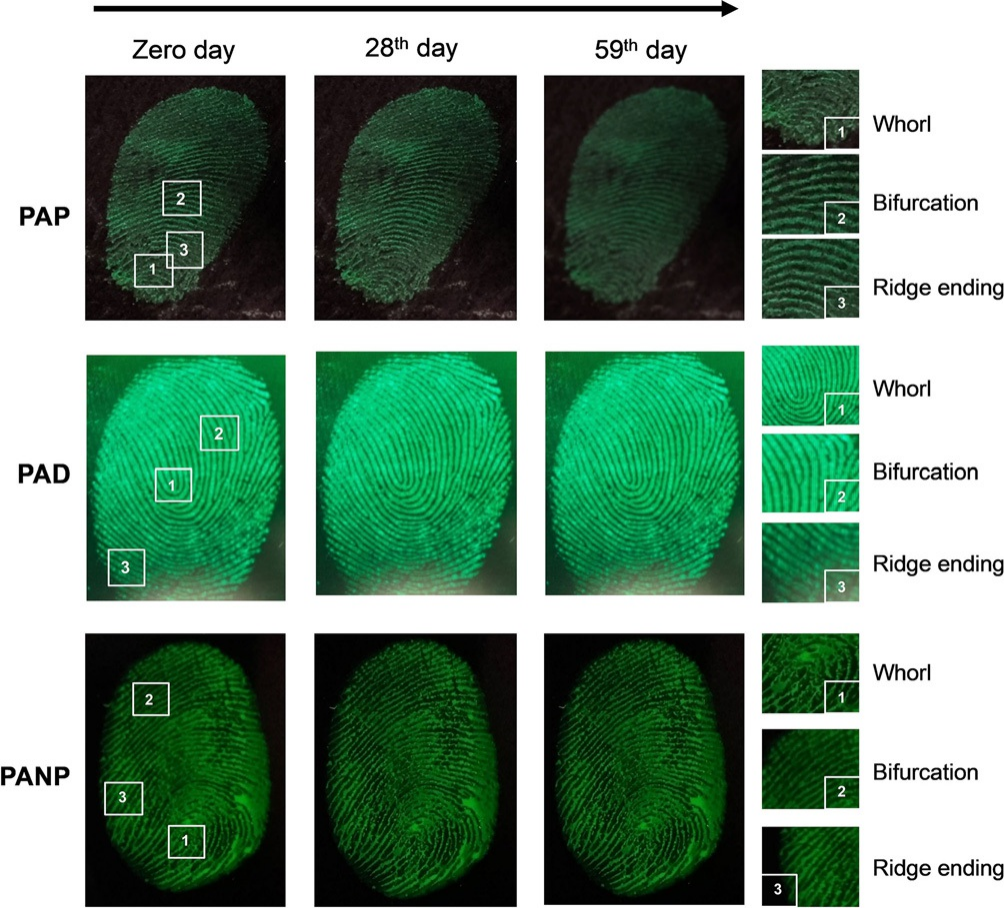
Photographs of fingerprints on smooth plastic surfaces detected by means of **PAP**, **PAD** and **PANP**. Enlarged areas marked with numbers are given on the right.

Importantly, relatively weak fluorescence was observed when thump‐printed fingerprints were rinsed with organic solvents such as CH_2_Cl_2_, THF and acetone, since these solvents could wash off the oily fingerprint residue. In contrast, samples rinsed with water exhibited intense fluorescence. This observation further underpins hydrophobic interactions as the first developing mechanism for the tested FLMs in the absence of any additives.^49^, ^55^, ^56^

In summary, novel fluorescent labeled nanohybrids based on polyhedral oligomeric silsesquioxanes (POSS) linked to appropriate fluorophores (pyrene, dansyl or naphthalimide) have been synthesized, fully characterized and their photophysical properties have been investigated in detail using different solvents. In particular, **PAP**, **PAD** and **PANP** displayed adequate physical characteristics and convenient photostability in order to be successfully applied in fingerprinting detection. In fact, **PAP**, **PAD** and **PANP** were successfully used to visualize latent fingerprints on several surfaces, providing reasonable legibility pattern that satisfy the needs of fingerprinting identifications in forensic technology.

## Conflict of interest

The authors declare no conflict of interest.

## Supporting information

As a service to our authors and readers, this journal provides supporting information supplied by the authors. Such materials are peer reviewed and may be re‐organized for online delivery, but are not copy‐edited or typeset. Technical support issues arising from supporting information (other than missing files) should be addressed to the authors.

Supplementary
