# Supplementary material for: Fluorescent‐Labeled Octasilsesquioxane Nanohybrids as Potential Materials for Latent Fingerprinting Detection
Source: Chemistry. 2020 Sep 17;26(58):13142–6. doi: 10.1002/chem.202001908 (PMC7692944; doi:10.1002/chem.202001908)
Supplement: Supplementary file 1 — Supplementary [file CHEM-26-13142-s001.pdf]

Chemistry–A European Journal

Supporting Information

Fluorescent-Labeled Octasilsesquioxane Nanohybrids as Potential Materials for Latent Fingerprinting Detection

Enock O. Dare,*[a, b] Victoria Vendrell-Criado,[c] M. Consuelo Jiménez,[c] Raúl Pérez-Ruiz,[c] and David Díaz Díaz*[a, d, e]

Table of Contents

	page
**Experimental Procedures**	S3
**Table S1**. Quantitative synthetic data for FLMs under optimized conditions	S6
**Figure S1.** ^{1}H NMR spectrum of **PAP**.	S7
**Figure S2.** ^{1}H NMR spectrum of **PAD**.	S8
**Figure S3.** ^{1}H NMR spectrum of **PANA**.	S9
**Figure S4.** ^{1}H NMR spectrum of **PANP**.	S10
**Figure S5**. ^{29}Si-NMR (**A**) and mass (**B**) spectra of **PAP**, **PAD**, **PANA** and **PANP**.	S11
**Figure S6.** FTIR spectra of **PAP** and its precursors under “click” conditions.	S12
**Figure S7.** Absorption (0.01 mM) and emission (λ_{exc} = 350 nm) spectra together with emission decay traces (λ_{exc} = 340 nm, filter at 370 nm) of **AP** and **PAP** in different solvents.	S13
**Figure S8.** Absorption (0.04 mM) and emission (λ_{exc} = 340 nm) spectra together with emission decay traces (λ_{exc} = 340 nm, filter at 400 nm) of **AD** and **PAD** in different solvents.	S14
**Figure S9.** Absorption (0.01 mM) and emission (λ_{exc} = 400 nm) spectra together with emission decay traces (λ_{exc} = 340 nm, filter at 370 nm) of **ANP** and **PANP** in different solvents.	S15
**Figure S10.** Absorption (0.02 mM) and emission (λ_{exc} = 355 nm) spectra together with emission (λ_{exc} = 340 nm, filter at 370 nm) of **ANA** and **PANA** in different solvents.	S16
**Figure S11.** UV-Vis spectra of PAZ, AD, PAD and PAZ + AD in dichloromethane under air. All concentrations were fixed at 0.04 mM.	S16
**Table S2A.** Photophysical properties of **AP** and **PAP**	S17
**Table S2B.** Values of the emission lifetimes of **AP** and **PAP** in different solvents	S17
**Table S3A.** Photophysical properties of **AD** and **PAD**	S18
**Table S3B.** Values of the emission lifetimes of **AD** and **PAD** in different solvents	S18
**Table S4A.** Photophysical properties of **ANP** and **PANP**	S19
**Table S4B.** Values of the emission lifetimes of **ANP** and **PANP** in different solvents.	S19
**Table S5.** Photophysical properties of **ANA** and **PANA**	S20
**Figure S12.** Proposed mechanism for the formation of excimer in **PAP**.	S21
**Figure S13.** Design of **PANP** fulfilling “receptor1-fluorophore-receptor2” model.	S21
**Figure S14.** Absorption and emission profile of alkynyl dye substrate (**AP**, **AD** and **ANP**) after 60 h monochromatic irradiation for photostability studies.	S22
**Figure S15.** Images of fingerprints detection on phone (a, b) and glass surfaces (c, d).	S23
**Additional Supplementary Figures**	S24
**Figure S16.** ^{1}H NMR spectrum of **AP**.	S24
**Figure S17.** 1H NMR spectrum of **ANA**.	S24
**Figure S18.** ^{1}H NMR spectrum of **ANP**.	S25
**Figure S19.** ^{1}H NMR spectrum of **AD.**	S25
**Author Contributions**	S26

Experimental Procedures

General remarks

^{1} H NMR spectra were recorded on a Bruker Avance 300 or 400 MHz spectrometer in $CDCl_3$ or d-DMSO. The residual undeuterated solvent signal was used as reference, relative to the tetramethylsilane signal. ^{13}C NMR were recorded on a Bruker Avance 300 or 400 MHz (respective resonance frequency: 75 and 101 MHz) under broadband ^{1} H decoupling in $CDCl_3$ or d-DMSO. ^{29}Si NMR were recorded in the same vein. The residual undeuterated solvent signal was used as reference, relative to the tetramethylsilane signal. ^{1} H NMR and ^{13}C NMR data were reported as follows: Chemical shifts were reported in the δ scale relative to residual $CDCl_3$ (7.26 ppm) and d-DMSO (2.50 ppm) for ^{1}H NMR and to the central line of $CDCl_3$ (77.16 ppm) and DMSO-d_6 (39.52 ppm) for ^{13}C NMR. FTIR spectra were obtained with an Agilent Technologies Cary 630 FTIR spectrometer equipped with Golden Gate Diamond ATR (attenuated total reflection. All reactions were monitored by thin-layer chromatography using Merck silica gel plates 60 F254; visualization was accomplished with short-wavelength UV light (254 nm) and/or staining with appropriate stains (anisaldehyde, orthophosphomolybdic acid). Standard flash chromatography was performed using Macherey-Nagel silica gel of a particle size 40−63 μm. 3-chloropropyl)hepta(*i*-butyl) octasilsesquioxane and *N*,*N*,*N*′,*N*″,*N*″-Pentamethyldiethylenetriamine (PMDETA) were purchased from Sigma-Aldrich. All other commercially available reagents and solvents were used without further purification.

Photophysical characterization

Absorption measurements

Steady state absorption spectra were recorded in a JASCO V-630 spectrophotometer. Quartz cells with 1 cm optical path length and 3 ml of capacity were employed. Molar coefficient extinction was determined according to the Lambert-Beer lay:

$$\mathrm{Abs} = \mathrm{C} \cdot \varepsilon \cdot \mathrm{L}$$

where, Abs is the absorbance of sample, C concentration, and L the optical path length of the cuvette.

Fluorescence experiments

Emission spectra were recorded on a JASCO FP-8500 spectrofluorometer system, provided with a monochromator in the wavelength range of 200–850 nm. From the intersection between normalized excitation and emission spectra the singlet energy was determined. Fluorescence quantum yields were determined using 9,10-dimethylantracene as standard (0.95, ETOH). Experiments were performed at 22 °C.

$$\phi_F = \frac{A_i}{A_{std}} \cdot \frac{Abs_{std}}{Abs_i} \cdot \frac{n}{n_{std}} \phi_{F(std)}$$

where, A_i is the fluorescence area of the sample, A_{std} is the fluorescence area of standard, Abs and Abs_{std} corresponds to the absorbance intensity at excitation wavelength of the sample and standard, respectively, and n is the refraction index of the solvent employed. Fluorescence lifetimes were recorded on a PTI (Photon Technology International) fluorometer which includes a pulsed LED excitation source, a sample holder and a lifetime detector. For lifetime analysis, EasyLife X software was used. The employed LEDs source were 340 or 375 nm.

Photostability studies

The three FLMs (**PAP**, **PAD** and **PANP**) and their respective alkynyl dye precursors (**AP**, **AD**, **ANA** and **ANP**) in THF, were irradiated with a monochromatic light for 60 h and photostability was monitored by absorption and emission at specific time intervals. A conservative monochromatic light irradiation of the samples was equally employed and the absorption profiles were monitored. For this, samples (1.0 x 10^{-5} M) were irradiated with a Xenon arc lamp at irradiances of 0.039 W/cm^2 and 0.052 W/cm^2.

Fingerprint development and imaging

As far as this study is concerned, fingermarks were collected from 3 voluntary donors (50 years old, 27 years old and 14 years old) and deposited on various substrate surfaces (plastic, glass bottle, leather and Nokia phone surface). The procedure involved the following steps: (1) Thump fingerprint by pressing the fingerprint on the desire surface; (2) immersion of the surface in the suitable fluorescent labeled nanohybrid (**PAP**, **PAD** or **PANP**) solution for 5 to 10 min; (3) rinse the surface with water to obtained the developed fingerprint; (4) fingerprint imaging using a digital camera exposing the fingerprint under UV light (365 nm), and (5) transfer of the image to the computer for further analysis.

Synthesis of compounds

Synthesis of alkynyl fluorophores

Ethynyl pyrene (**AP**), Propargyl dansyl (**AD**) and propargyl bromo naphthalic anhydride (**ANA**) were prepared following literature procedure: **AP** [ref. 23 in main text], **AD** [ref. 24 in main text] and **ANA** [ref. 25 in main text]. ^{1}H and ^{13}C NMR spectra for these alkynylated fluorophores conform to the values in the literature.

*Synthesis of propargyl naphthalic anhydride piperazine (**ANP**)*

To a solution of **ANA** (0.5 g, 1.59 mM) in 2-methoxylethanol (20 mL) was added piperazine (0.150 g, 1.74 mM). The mixture was refluxed for 16 h. The yellowish green solid (0.41 g) obtained was washed with ethanol providing the desired product in 82% yield.
^{1}H NMR (300 MHz, DMSO-d_6): δ = 1.24 (s, 1H, piperazine terminal NH), 3.14 (s, 1H, -CH_2-C≡CH), 3.40-3.49 (quin, overlapped 8H of piperazine, 4 x CH_2), 4.76 (-N-CH_2-C≡CH,), 7.4, 7.8, 8.5, 8.6, 8.8 (all d, 5H, CH in naphthalimide aromatic); ^{13}C NMR (125 MHz, $CDCl_3$) ppm: 29.1, 40.2, 50.0, 72.2, 80.0, 57.6, 64.5, 116.1, 116.9, 122.2, 125.6, 126, 133,8, 155.3, 167.4 173.9; MS (API-ESI) m/z: 320.08 $[M+H]^+$; Elemental analysis: calculated for $C_{19}H_{17}N_3O_2$ (MW 319.36) C 71.47, H 5.33, N 13.16; found C 71.91, H 5.62, N 13.38 %.

*Synthesis of 3-azidopropyl hepta(i-butyl) POSS (**PAZ**)*

Following a literature procedure [ref. 22 in main text], the major precursor, **PAZ** was synthesized: Under a dry nitrogen atmosphere, a solution mixture of dry DMF (70 mL), dry THF (20 mL), and 3-chloropropyl)hepta(i-butyl) POSS (2.0 g, 2.24 mmol) was added with 3 equivalents of sodium azide (6.71 mmol, 0.436 g). The solution mixture was kept stirring at 70 ºC for 24 h and the reaction was cooled to RT. The reaction solution was quenched by pouring the mixture on crushed ice (80 g). The obtained precipitate was filtered, dried under vacuum, and recrystallized from a mixture THF/methanol to obtain the desired compound as a colorless compound (1.43 g, 2.09 mmol, 81% yield).
^{1}H NMR (300 MHz, $CDCl_3$): δ = 3.28 (t, 2H), 1.90 (m, 7H), 1.75 (quin, 2H), 0.97 (d, 42H), 0.70 (overlapped, 2H), 0.63 (m, 14H). ^{13}C NMR (125 MHz, $CDCl_3$): δ = 52.88, 25.66, 25.69, 23.89, 23.84, 22.46, 22.41, 9.31. ^{29}Si NMR (99 MHz, $CDCl_3$): δ = -67.55, -68,17.

*Synthesis of (3-pyrenetriazolylpropyl) hepta(i-butyl) POSS (**PAP**)*

Under a dry nitrogen atmosphere, **AP** (0.085 g, 0.38 mM) was added to solution of **PAZ** (0.324 g, 0.36 mM) in DMF/THF (10 mL) (1:1). Other solvents systems were attempted for comparison. CuBr (2.5 mol%) and PMDETA (2.5 mol%) were successively added. After 18 h reaction, 0.02 M EDTA was added and extracted with DCM. Organic phase was further washed with deionized water and Na_2SO_4 added. After filtration and concentration in a rotary evaporator, further purification was achieved in a column chromatography (Hexane: Ethyl acetate, 30%) to afford the desired product as a greenish-yellow solid (0.077 g, 91% yield).
^{1}H NMR (300 MHz, $CDCl_3$): δ = 0.63 (d, 14H, Si-$\underline{CH_2}$CH(CH_3)$_2$, 0.73 (t, 2H, Si-$\underline{CH_2}CH_2$-), 0.95 (d, 42H, Si-CH_2CH($\underline{CH_3}$)$_2$), 1.81 (m, 7H, Si-$CH_2\underline{CH}$(CH_3)$_2$), 2.22 (m, 2H, Si-$CH_2\underline{CH_2}CH_2$-), 4.55 (t, 2H, Si-$CH_2CH_2\underline{CH_2}$N-), 7.9 (s, 1H, in 1,2,3-triazole), 8.0-8.25, 8.7(s, d, 9H, CH in pyrene aromatic); ^{13}C NMR (125 MHz, $CDCl_3$): δ (ppm): 10.1, 22.4, 23.1, 25.1, 26.3, 47.1, 53.2, 121.8-144.1; ^{29}Si NMR (59.6 MHz, $CDCl_3$): δ (ppm): -67.8, -68.2 (Si-O-Si); FT-IR (cm^{-1}): *v*(CH) 2992-2891; δ (CH) 1374-1211; *v*(C=C) 1658; *v*(Si-O-Si) 1134-1022; MS (API-ESI) m/z: 1126.4 $[M+H]^+$. Elemental analysis: calculated for $C_{49}H_{79}N_3O_{12}Si_8$ (MW 1126.86) C 52.27, H 7.01, N 3.73; found C 52.31, H 6.99, N 3.68 %.

*Synthesis of (3-dansyltriazolylpropyl) hepta(i-butyl) POSS (**PAD**)*

To a solution of **AZ** (0.070 g, 7.8 x 10^{-2} mM) in 10 mL DMF/THF (1:1) was added **AD** (0.023 g, 8.0 x 10^{-2} mM), CuBr (2.5 mol%) and PMDETA (2.5 mol%) was successively added. The mixture was stirred at RT. Solvent was removed by rotary evaporator and

redissolved in DCM. 0.02 M EDTA was added while extraction proceeded in DCM, dried (Na_2SO_4) and evaporated at reduced pressure. The crude was purified using column chromatography (hexane/EtOAc 10:2) to afford 0.050 g (72%) greenish-yellow product. Reaction optimization was achieved while varying solvents and catalyst. Note: Typically, all materials obtained after the "click" reaction are passed through Celite and then washed with 0.02 M EDTA solution overnight, replacing the solution 3-4 times during this period until no more blue color due to the extraction of metal ions is observed.
^{1}H NMR (300 MHz, $CDCl_3$): δ = 0.61 (overlapped 14H, Si-$\underline{CH_2}$CH(CH_3)$_2$, 0.62 (overlapped 2H, Si-$\underline{CH_2}$$CH_2$-), 0.99 (d, 42H, Si-$\underline{CH_2}$CH($\underline{CH_3}$)$_2$), 1.85 (overlapped 7H, Si-$CH_2$$\underline{CH}$($CH_3$)$_2$), 1.88 (overlapped 2H, Si-$CH_2$$\underline{CH_2}$$CH_2$-), 2.90 (s, 6H, Ar-N-($\underline{CH_3}$)$_2$), 4.23 (overlapped 2H, Si-$CH_2$$CH_2$$\underline{CH_2}$N-), 4.24 (overlapped 2H, -triazole-$\underline{CH_2}$-NH-) 7.26 (s, 1H, in 1,2,3-triazole), 8.1 (s, 1H, -triazole-CH_2-$\underline{NH}$-) 7.23, 7.5, 7.6, 8.25, 8.60 (all d, 6H, CH in dansyl aromatic); ^{13}C NMR (125 MHz, $CDCl_3$): δ (ppm): 9.8, 22.5, 23, 26, 39, 45, 117.1-132.6; ^{29}Si NMR (59.6 MHz, $CDCl_3$): δ (ppm): -67.93, -68.23 (Si-O-Si); FT-IR (cm^{-1}): *v*(CH) 2932.6; (CN) 1646; (NCO) 1644.7 *v*(C=C-Ar) 1741.9; *v*(Si-O-Si) 1161-1020; MS (API-ESI) m/z: 1188.40 [M+H]$^+$. Elemental analysis: calculated for $C_{46}H_{85}N_5O_{14}SSi_8$ (MW 1188.95) C 46.50, H 7.16, N 5.90; found C 46.77, H 7.19, N 5.76 %.

*Synthesis of (3-bromonaphthalic anhydride triazolylpropyl) hepta(i-butyl) POSS (**PANA**)*

To a colloidal solution of **PN** (0.070 g, 0.220 mM) in 10 mM (DMF/THF 1:1) was added **AZ** (0.2 g, 0.220 mM). This was followed by the successive addition of CuBr (2.5 mol%) and PMDETA (2.5 mol%). The colloidal mixture was stirred overnight at RT. The light-yellow precipitate was filtered and washed with methanol to obtain the desired product as a light-yellow product (0.160 g, 70 %). Reaction optimization was subsequently carried out in varying solvents and catalysts. Note: Typically, all materials obtained after the "click" reaction are passed through Celite and then washed with 0.02 M EDTA solution overnight, replacing the solution 3-4 times during this period until no more blue color due to the extraction of metal ions is observed.
^{1}H NMR (300 MHz, $CDCl_3$): δ = 0.61 (d, 14H, Si-$\underline{CH_2}$CH(CH_3)$_2$, 0.63 (overlapped 2H, Si-$\underline{CH_2}$$CH_2$-), 0.98 (d, 42H, Si-$\underline{CH_2}$CH($\underline{CH_3}$)$_2$), 1.82 (m, 7H, Si-$CH_2$$\underline{CH}$($CH_3$)$_2$), 1.91 (m, 2H, Si-$CH_2$$\underline{CH_2}$$CH_2$-), 4.27 (t, 2H, Si-$CH_2$$CH_2$$\underline{CH_2}$N-), 5.5 (s, 2H, triazole-CH_2-N) 7.6 (s, 1H, in 1,2,3-triazole), 7.8, 8.1, 8.45, 8.6, 8.7 (all d, 5H, CH in naphthalimide aromatic); ^{13}C NMR (125 MHz, $CDCl_3$): δ (ppm): 9.5, 22.4, 29.5, 47.1, 53.2, 70.1 121.3, 122.6, 126.1, 130.2-134.6; ^{29}Si NMR (59.6 MHz, $CDCl_3$): δ (ppm): -67.9, -68.3 (Si-O-Si); FT-IR (cm^{-1}): *v*(CH) 2932.6; (CN) 1696; (NCO) 1654.7 *v*(C=C-Ar) 1588.9; *v*(Si-O-Si) 1150-1022; MS (API-ESI) m/z: 1215.28 [M+H]$^+$. Elemental analysis: calculated for $C_{46}H_{77}BrN_4O_{14}Si_8$ (MW 1214.72) C 45.52, H 6.35, N 4.62; found C 45.59, H 6.35, N 4.61 %.

*Synthesis of (3-piperazine-naphthalic anhydride triazolylpropyl) hepta(i-butyl) POSS (**PANP**): Method 1*

ANP (0.020 g, 6.3 x 10^{-2} mM) was added to stirring **AZ** (0.050 g, 5.6 x 10^{-2} mM) in 10 mL DMF/THF (1:1). The resulting mixture was stirred at RT overnight. The crude precipitate, greenish-yellow in colour, was washed with methanol and dried to give the desired product (0.034 g, 68%). Optimization was further carried out in varying solvents system and catalysts. Note: Typically, all materials obtained after the "click" reaction are passed through Celite and then washed with 0.02 M EDTA solution overnight, replacing the solution 3-4 times during this period until no more blue color due to the extraction of metal ions is observed.
^{1}H NMR (300 MHz, $CDCl_3$): δ = 0.61 (overlapped 14H, Si-$\underline{CH_2}$CH(CH_3)$_2$, 0.62 (overlapped 2H, Si-$\underline{CH_2}$$CH_2$-), 0.98 (d, 42H, Si-$\underline{CH_2}$CH($\underline{CH_3}$)$_2$), 1.30 (s, 1H, -piperazine-$\underline{NH}$) 1.85 (m, 7H, Si-$CH_2$$\underline{CH}$($CH_3$)$_2$), 1.90 (m, 2H, Si-$CH_2$$\underline{CH_2}$$CH_2$-), 3.51 (s, 4H, 2 x piperazine CH_2) 3.90 (s, 4H, 2 x piperazine CH_2) 4.27 (t, 2H, Si-$CH_2$$CH_2$$\underline{CH_2}$N-), 5.6 (s, 2H, triazole-CH_2-N) 7.6 (s, 1H, in 1,2,3-triazole), 7.85, 8.1, 8.45, 8.6, 8.7 (all d, 5H, CH in naphthalimide aromatic); ^{13}C NMR (125 MHz, $CDCl_3$): δ (ppm): 12.1, 21.0, 26.3, 30, 43, 46, 54, 118-143); ^{29}Si NMR (59.6 MHz, $CDCl_3$): δ (ppm): -67.92, -68.25 (Si-O-Si); FT-IR (cm^{-1}): *v*(CH) 2932.6; (CN) 1696; (NCO) 1654.7 *v*(C=C-Ar) 1588.9; *v*(Si-O-Si) 1150-1022; MS (API-ESI) m/z: 1219.44 [M+H]$^+$. Elemental analysis: calculated for $C_{50}H_{86}N_6O_{14}Si_8$ (MW 1219.44) C 40.05, H 5.74, N 5.61; found C 40.59, H 6.46, N 5.93 %.

*Synthesis of (3-piperazine-naphthalic anhydride triazolylpropyl) hepta(i-butyl) POSS (**PANP**): Method 2*

PANA (0.035 g, 2.9 x 10^{-2} mM) was added to piperazine (0.003 g, 3.5 x 10^{-2} mM). The resulting mixture was heated to 90 °C and stirred overnight. The obtained greenish-yellow precipitate was washed with methanol to give **PANP** (0.0154 g, 44% yield). The characterization data were identical to those obtained using Method 1.

Table S1. Quantitative synthetic data for FLMs under optimized conditions

					Yield [%][a]				
Entry]	Catalyst	Ligand	Solvent (v/v ratio)	t (h)	**PAP**	**PAD**	**PANA**	[**PANP**][b]	[**PANP**][c]
1	CuBr/PMDETA	PMDETA	DMF	18	41	39	46	16	
**2**	**CuBr/PMDETA**	**PMDETA**	**THF/DMF (1/1)**	**18**	**81**	**74**	**91**	**68**	
3	CuBr		THF/DMF (1/1)	36	66	51	40	22	
4	$CuSO_4$/NaAsc		CH_2Cl_2/H_2O (1/1)	36	8	7	12		
5	$CuSO_4$/NaAsc		THF/H_2O (2/1)	28	7	8	10	62	
6	CuBr/PMDETA	PMDETA	DMSO/THF	24	51	42	18		
7			2-methoxy ethanol	18					44

[a] Isolated yield. [b] CuAAC procedure with **ANP**. [c] Aminolysis of **PANA** with piperazine in the presence of 2-methoxyl ethanol as solvent and without catalyst.

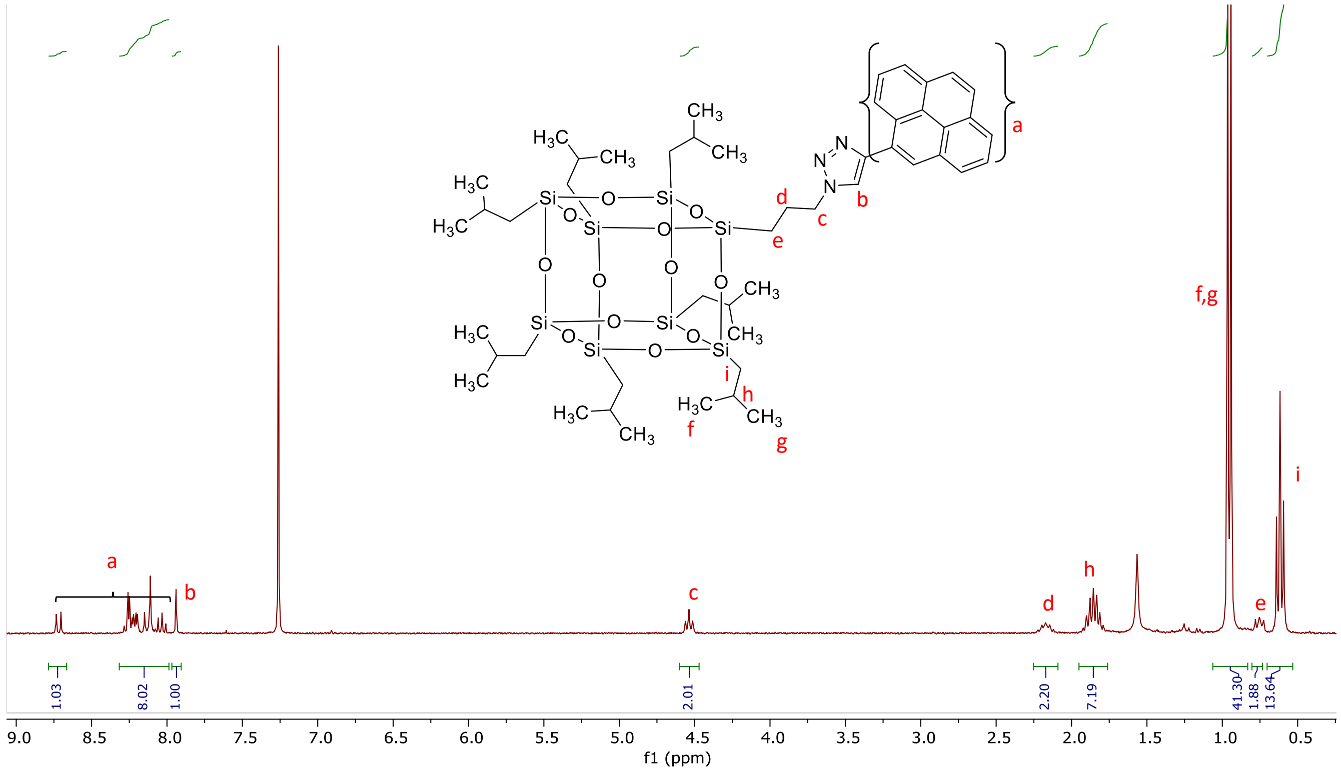

Figure S1. ^{1}H NMR spectrum of **PAP**.

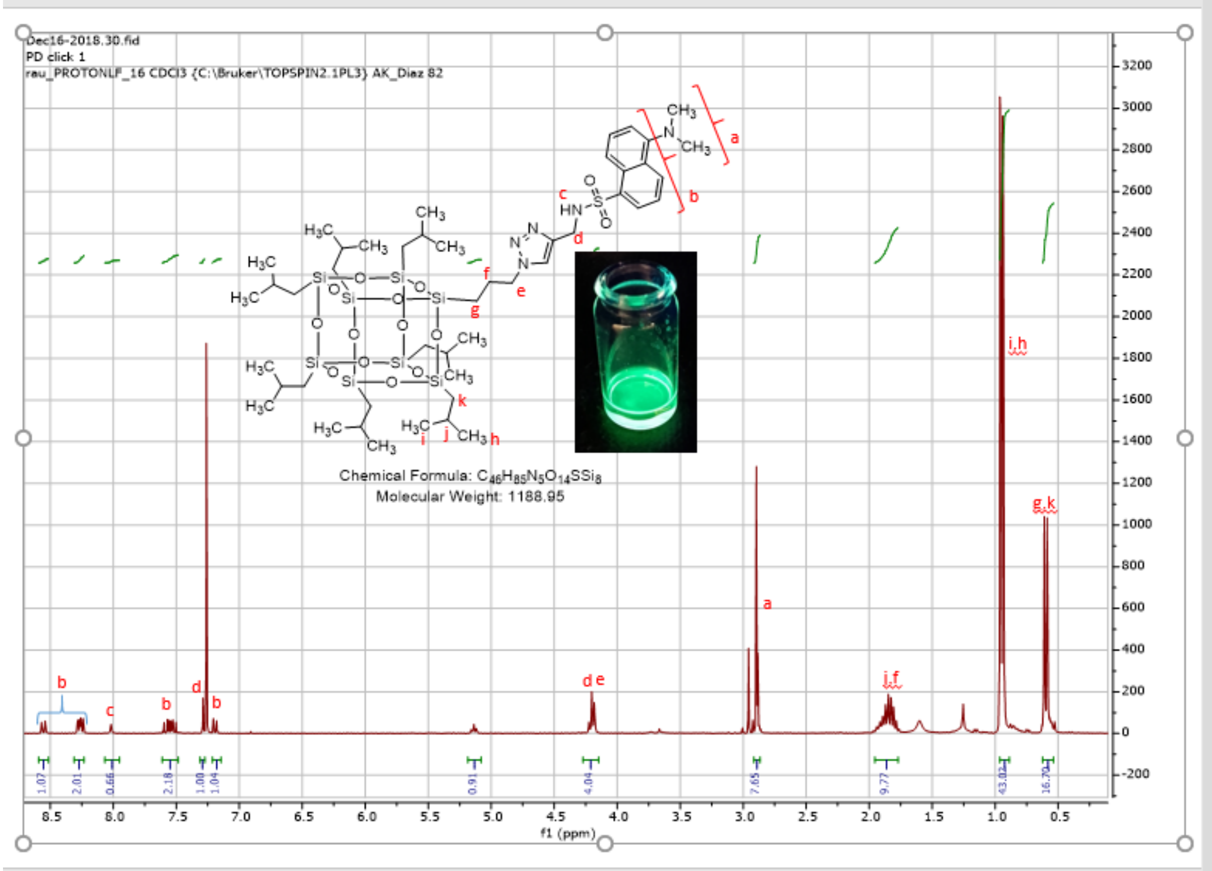

Figure S2. ^{1}H NMR spectrum of **PAD**.

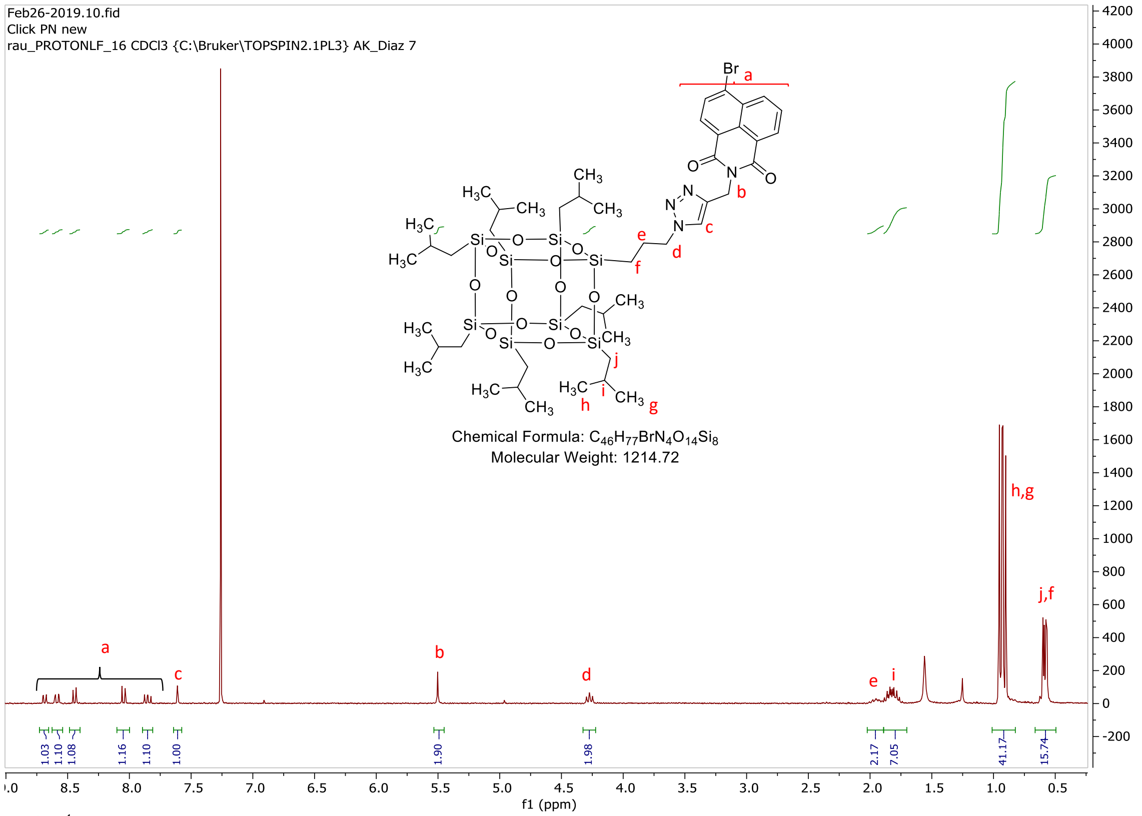

Figure S3. ^{1}H NMR spectrum of **PANA**.

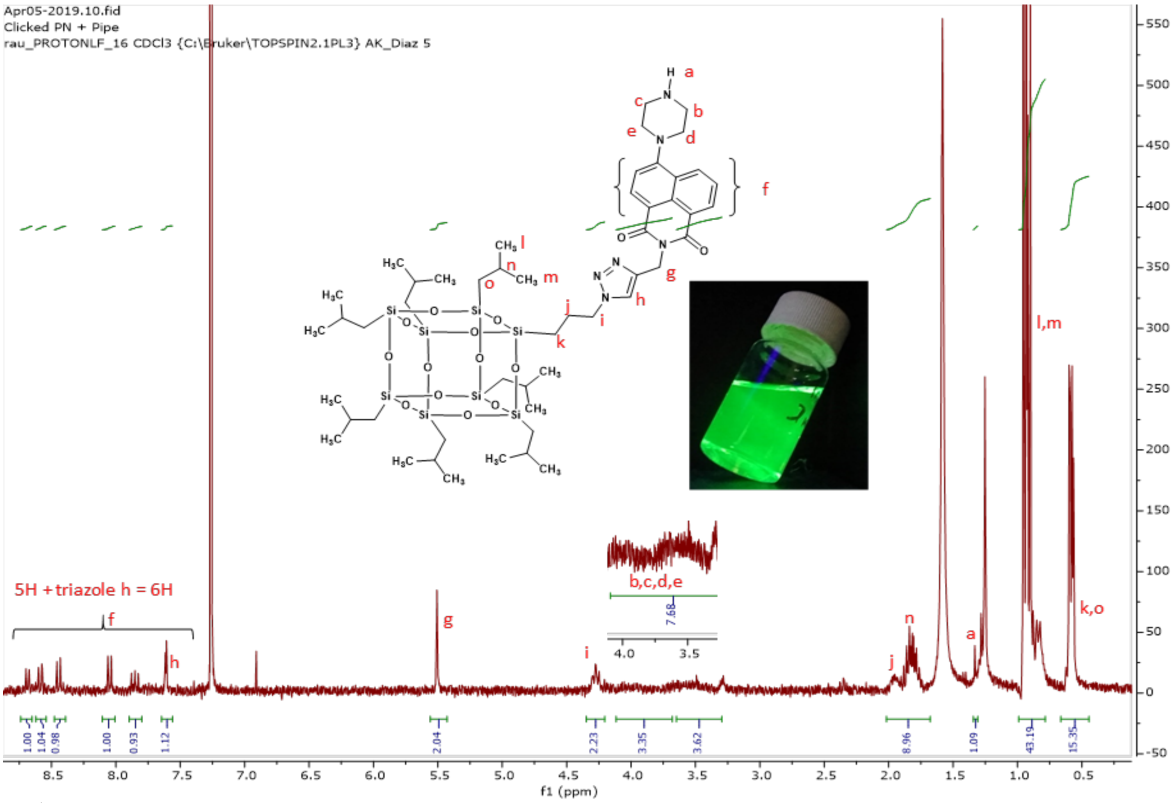

Figure S4. ^{1}H NMR spectrum of **PANP**.

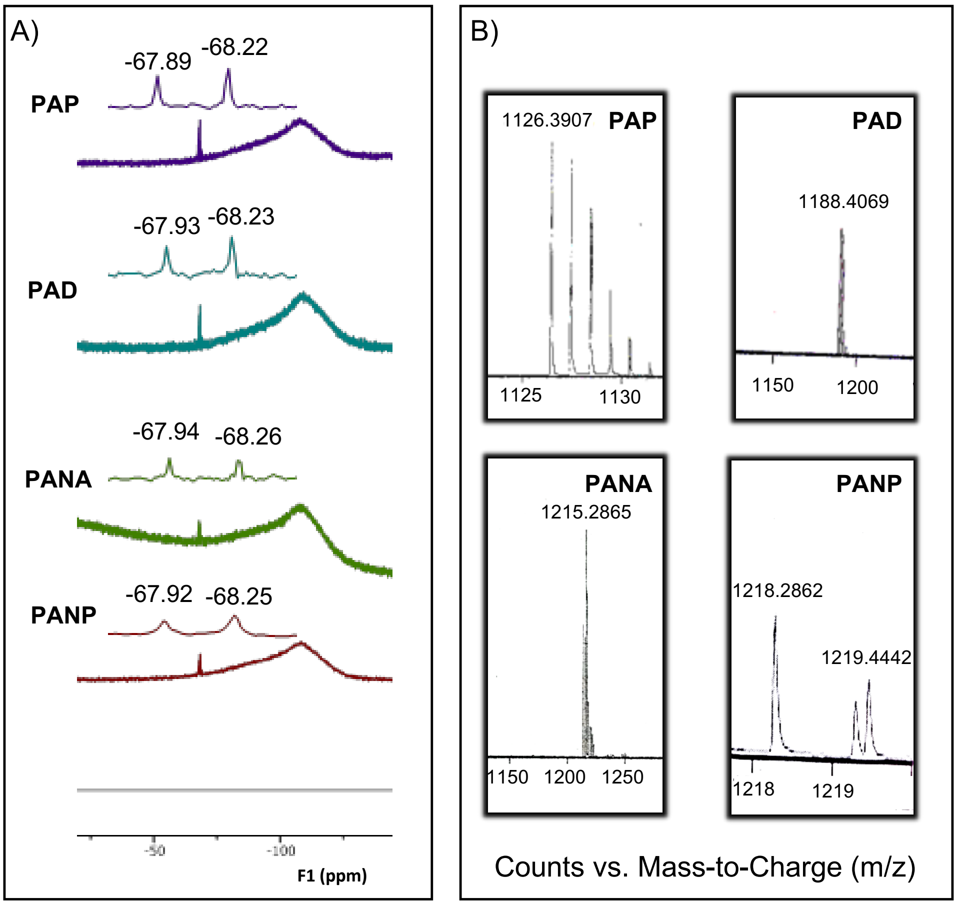

Figure S5. ^{29}Si-NMR (**A**) and mass (**B**) spectra of **PAP**, **PAD**, **PANA** and **PANP**.

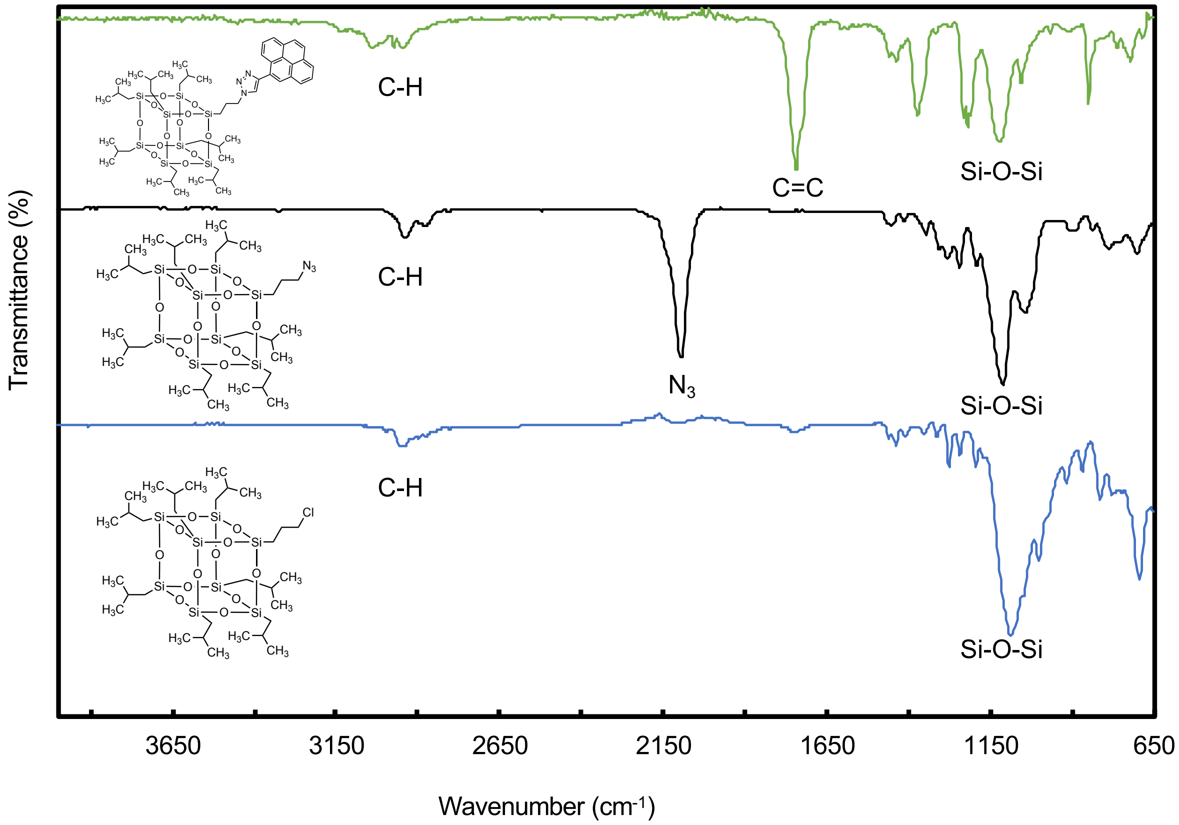

Figure S6. FTIR spectra of **PAP** and its precursors under “click” conditions.

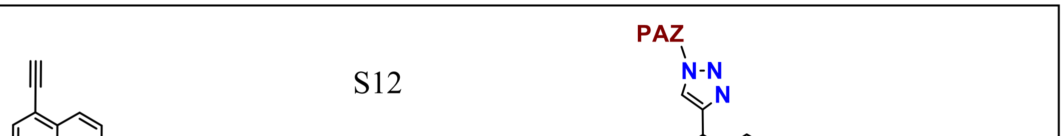

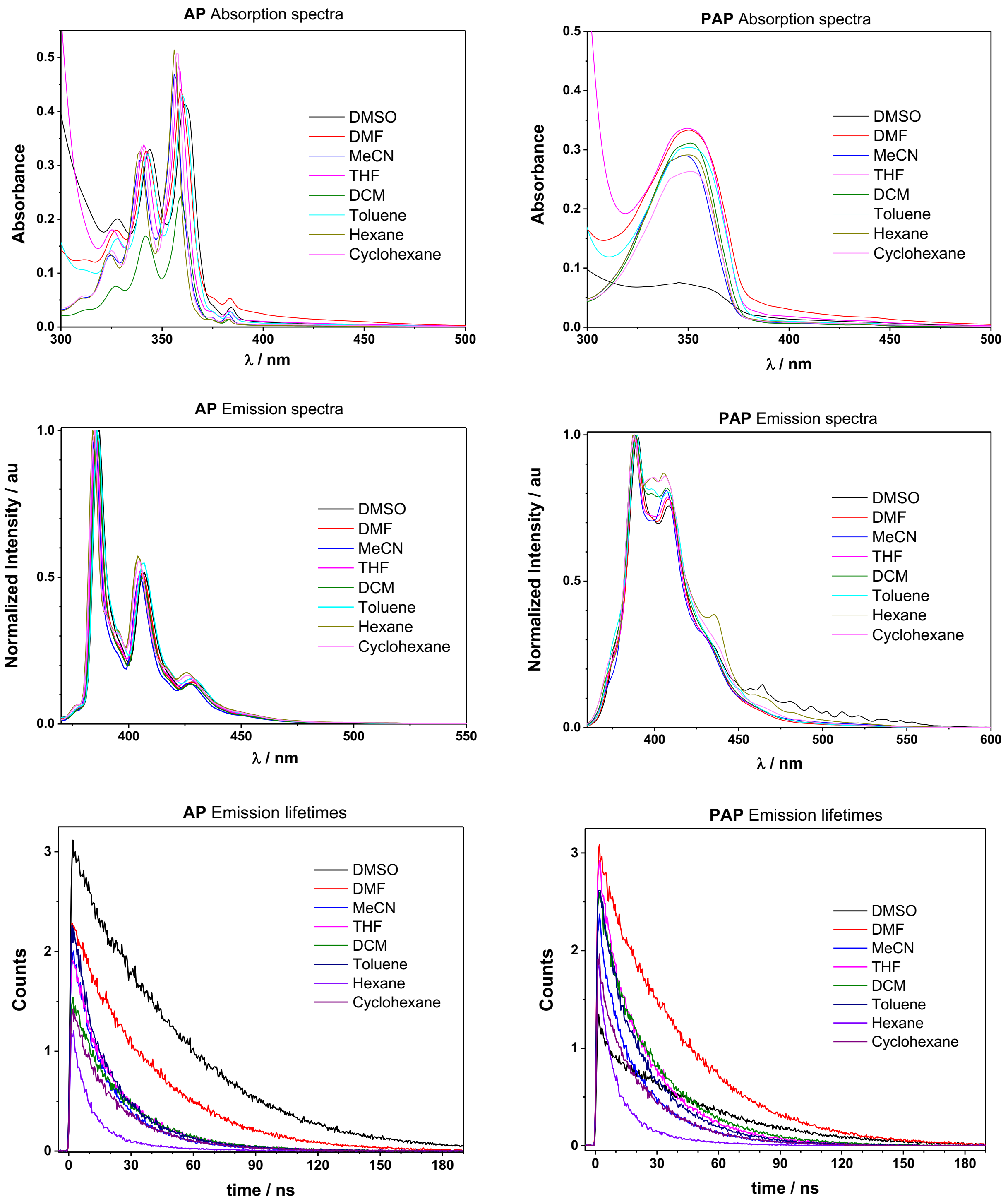

Figure S7. Absorption (0.01 mM) and emission (λ_{exc} = 350 nm) spectra together with emission decay traces (λ_{exc} = 340 nm, filter at 370 nm) of **AP** and **PAP** in different solvents.

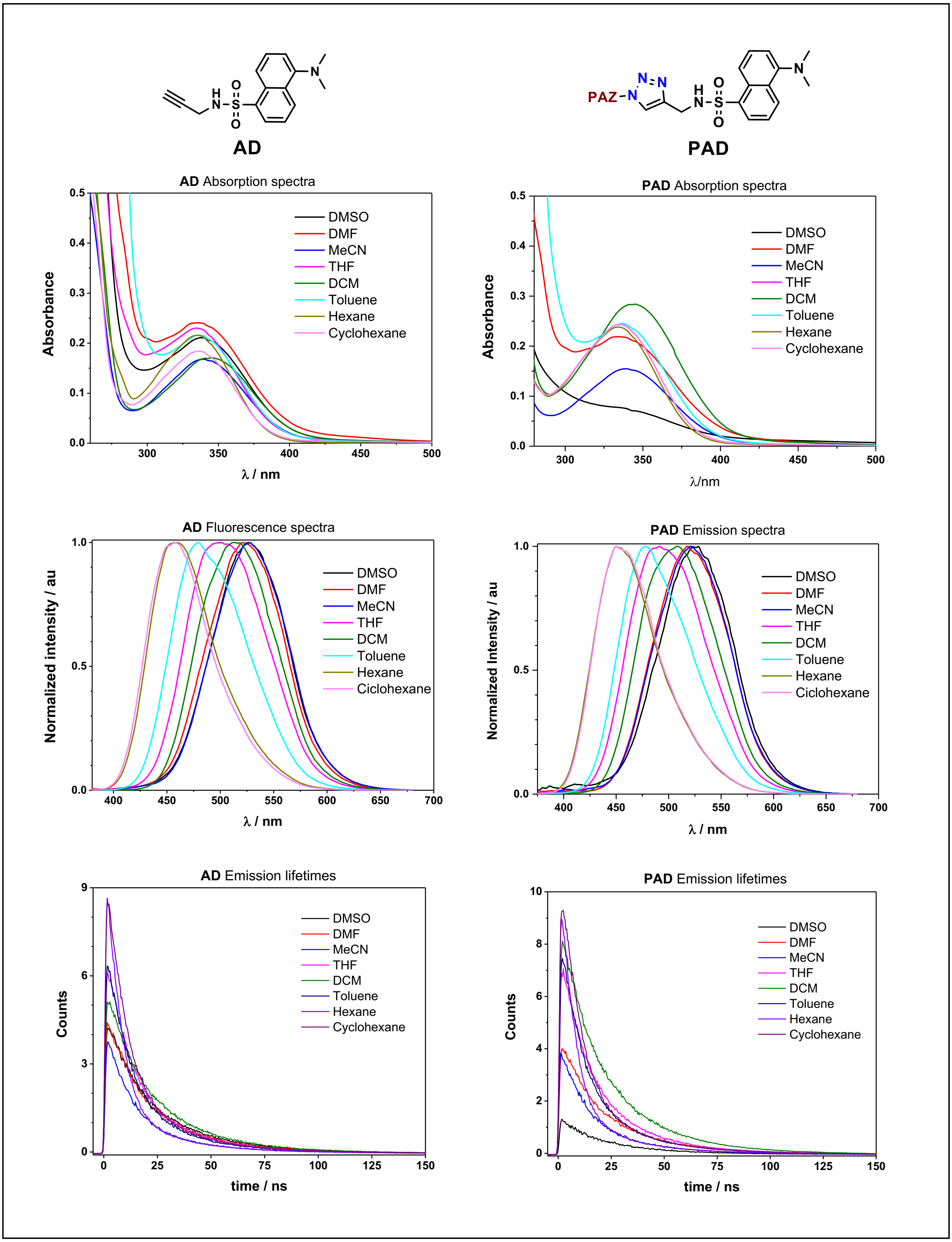

Figure S8. Absorption (0.04 mM) and emission (λ_{exc} = 340 nm) spectra together with emission decay traces (λ_{exc} = 340 nm, filter at 400 nm) of **AD** and **PAD** in different solvents.

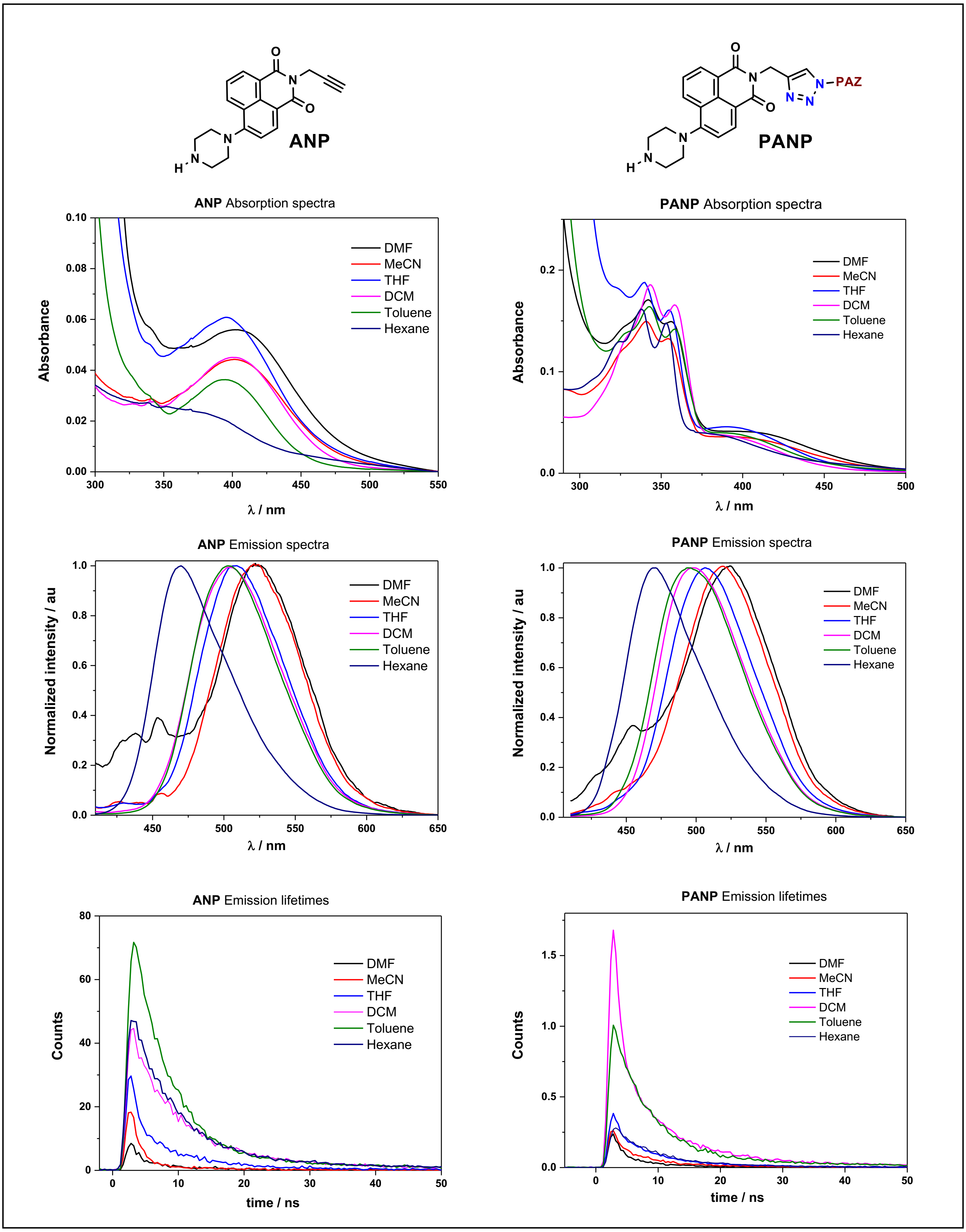

Figure S9. Absorption (0.01 mM) and emission (λ_{exc} = 400 nm) spectra together with emission decay traces (λ_{exc} = 340 nm, filter at 370 nm) of **ANP** and **PANP** in different solvents.

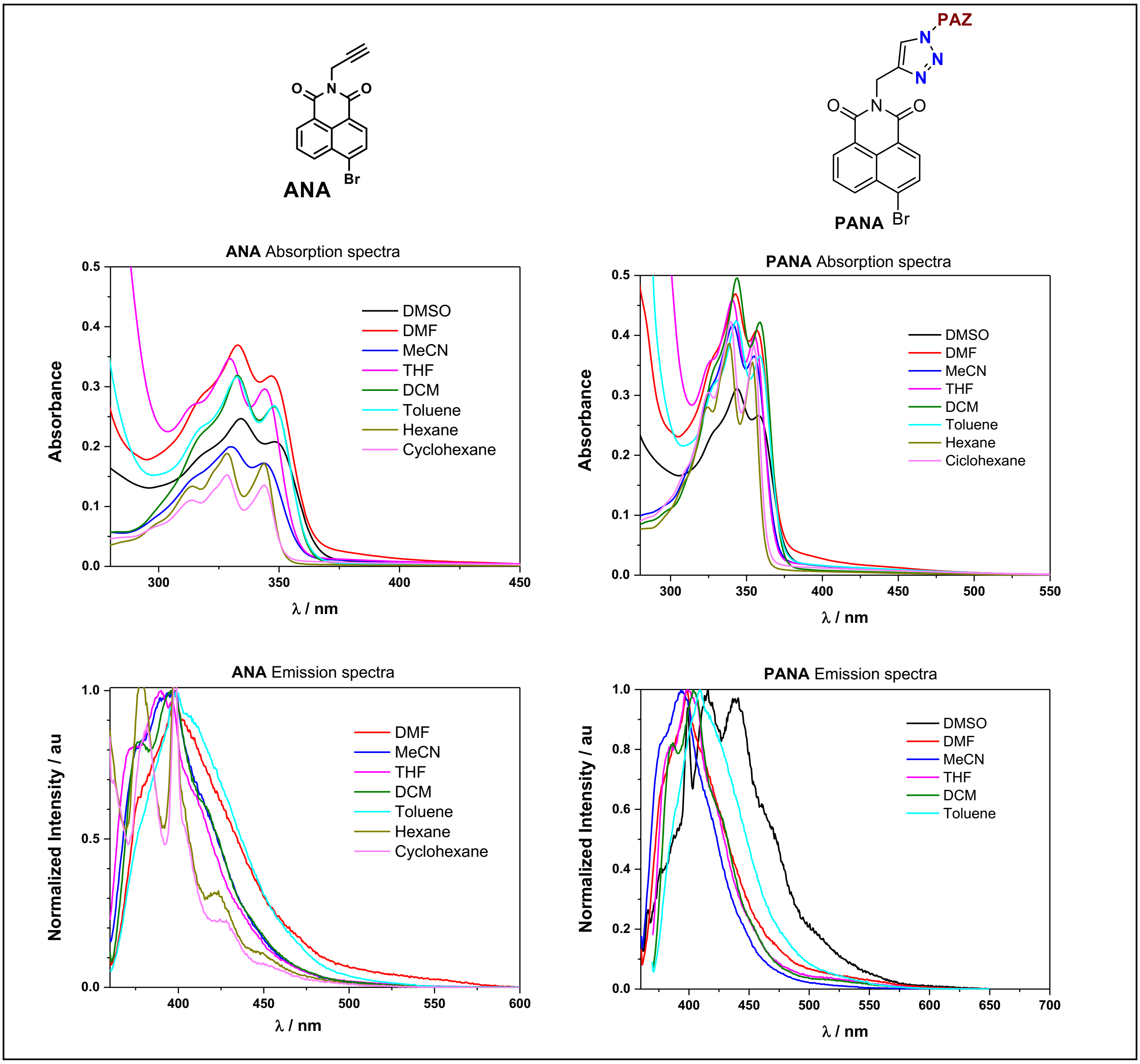

Figure S10. Absorption (0.02 mM) and emission (λ_{exc} = 355 nm) spectra together with emission (λ_{exc} = 340 nm, filter at 370 nm) of **ANA** and **PANA** in different solvents.

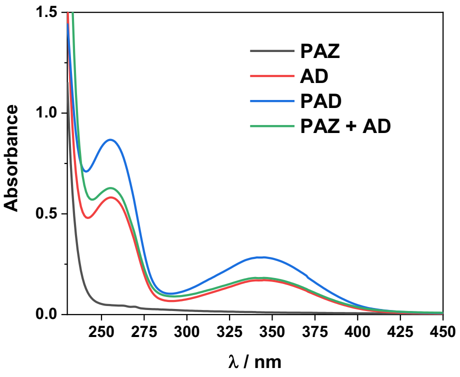

Figure S11. UV-Vis spectra of PAZ, AD, PAD and PAZ + AD in dichloromethane under air. All concentrations were fixed at 0.04 mM.

Table S2A. Photophysical properties of **AP** and **PAP**

	**AP**								**PAP**							
**Solvents**	$^a\lambda_{abs\ max}$	$^a\lambda_{em\ max}$	$^b\varepsilon$	^{c}Stokes	^dE_S	ϕ_F	ϕ_{nrad}	^ek_F x10^7	$^a\lambda_{abs\ max}$	$^a\lambda_{em\ max}$	$^b\varepsilon$	^{c}Stokes	^dE_S	ϕ_F	ϕ_{nrad}	^ek_F x10^7
**DMSO**	327, 344, 361	386, 406, 429	41284	1794	75.4	0.61	0.39	1.1	351	390, 409	7297	2849	76.2	0.69	0.31	1.2
**DMF**	328, 343, 359	386, 406, 428	44177	1794	75.8	0.29	0.71	0.7	350	389, 408	33330	2865	76.4	0.34	0.66	0.8
**ACN**	325, 339, 356	385, 405, 427	49890	2115	76.0	0.17	0.83	1.7	348	388, 407	28989	2962	76.8	0.14	0.86	2.5
**THF**	324,341, 358	384, 404, 426	48355	1891	75.8	0.14	0.86	1.4	349	388, 408	33639	2798	76.4	0.19	0.81	3.6
**DCM**	328, 343, 360	386, 406, 428	24235	1871	75.8	0.14	0.86	1.7	348	389, 407	31132	3029	76.6	0.22	0.78	3.9
**TOL**	326, 343,360	385, 405, 428	43064	1803	75.6	0.19	0.81	1.8	349	389, 408	30394	2652	76.4	0.20	0.80	2.7
**HEX**	324, 340, 357	386, 407, 429	51426	2105	76.2	0.17	0.83	2.5	351	388, 405	29136	2717	76.6	0.06	0.94	1.0
**CHX**	326, 341, 359	386, 405, 426	50686	1949	76.2	0.15	0.85	1.8	353	388, 405	26251	2798	76.8	0.16	0.84	4.4

^{a}in nm; ^{b}in $M^{-1}cm^{-1}$; ^{c}in cm^{-1}; ^{d}in kcal mol^{-1}; ^{e}in s^{-1}

Table S2B. Values of the emission lifetimes of **AP** and **PAP** in different solvents

**Solvents**	**Polarity**	**AP (τ_1; τ_2) / ns**	**PAP (τ_1; τ_2) / ns**
**DMSO**	7.8	54.9 ± 0.3 (100%) R^2 0.998	55.5 ± 0.5 (100%) R^2 0.996
**DMF**	6.4	38.8 ± 0.12 (100%) R^2 0.998	41.7 ± 0.2 (100%) R^2 0.998
**ACN**	5.8	9.9 ± 0.5 (97%); 24.0 ± 0.9 (3%) R^2 0.998	5.5 ± 0.2 (99%); 22.8 ± 0.2 (1%) R^2 0.998
**THF**	4.0	9.9 ± 0.7 (95%); 25.5 ± 0.7 (5%) R^2 0.998	5.2 ± 0.3 (99%); 25.1 ± 0.2 (1%) R^2 0.999
**DCM**	3.1	8.2 ± 1.4 (97%); 26.3 ± 0.5 (3%) R^2 0.997	5.6 ± 0.4 (99%); 28.7 ± 0.2 (1%) R^2 0.998
**TOL**	2.4	10.2 ± 0.6 (97%); 25.4 ± 1 (3%) R^2 0.998	7.2 ± 0.5 (99%); 24.7 ± 0.3 (1%) R^2 0.998
**HEX**	0.1	6.6 ± 0.1 (99%); 21.8 ± 1 (1%) R^2 0.998	5.8 ± 0.1 (99%); 19.8 ± 0.5 (1%) R^2 0.998
**CHX**	0.0	8.1 ± 0.8 (98%); 24.5 ± 0.5 (2%) R^2 0.997	3.6 ± 0.2 (99%); 24.1 ± 0.2 (1%) R^2 0.998

Table S3A. Photophysical properties of **AD** and **PAD**

	**AD**								**PAD**							
**Solvents**	$^{a}\lambda_{abs\ max}$	$^{a}\lambda_{em\ max}$	$^{b}\varepsilon$	^{c}Stokes	$^{d}E_S$	ϕ_F	ϕ_{nrad}	$^{e}k_F$ x10^7	$^{a}\lambda_{abs\ max}$	$^{a}\lambda_{em\ max}$	$^{b}\varepsilon$	^{c}Stokes	$^{d}E_S$	ϕ_F	ϕ_{nrad}	$^{e}k_F$ x10^7
**DMSO**	338	524	5272	10502	67.4	0.48	0.52	3.0	338	528	1872	10646	68.9	0.37	0.63	3.3
**DMF**	337	519	6012	10406	67.9	0.37	0.63	2.3	332	519	5419	10853	67.5	0.38	0.62	2.3
**ACN**	340	524	4195	10328	67.9	0.34	0.66	2.8	340	521	3867	10254	68.2	0.39	0.61	2.9
**THF**	334	497	5748	9820	66.9	0.55	0.45	4.0	335	491	6087	9436	69.2	0.62	0.38	4.8
**DCM**	345	512	4272	9454	66.9	0.65	0.35	4.6	345	508	7098	9384	67.3	0.67	0.33	4.2
**TOL**	336	482	5399	9015	69.1	0.59	0.41	5.0	337	477	6123	8709	69.0	0.65	0.35	5.6
**HEX**	335	455	5392	7872	71.6	0.57	0.43	7.5	334	450	5954	7768	71.9	0.55	0.45	7.2
**CHX**	336	454	4605	7647	71.5	0.93	0.07	8.9	335	450	6072	7501	71.7	0.80	0.2	7.9

^{a}in nm; ^{b}in $M^{-1}cm^{-1}$; ^{c}in cm^{-1}; ^{d}in kcal mol^{-1}; ^{e}in s^{-1}

Table S3B. Values of the emission lifetimes of **AD** and **PAD** in different solvents.

**Solvents**	**Polarity**	**AD (τ_1; τ_2) / ns**	**PAD (τ_1; τ_2) / ns**
**DMSO**	7.8	15.6 ± 0.7 (93%); 35.6 ± 2.3 (7%) R^2 0.999	11.1 ± 1.3 (93%); 28.3 ± 1.2 (7%) R^2 0.997
**DMF**	6.4	16.2 ± 0.5 (96%); 39.8 ± 3.2 (4%) R^2 0.999	16.0 ± 0.6 (95%); 36.6 ± 2.7 (5%) R^2 0.999
**ACN**	5.8	12.2 ± 0.2 (99%); 39.5 ± 2.0 (1%) R^2 0.999	13.5 ± 0.2 (99%); 42.4 ± 3.6 (1%) R^2 0.998
**THF**	4.0	13.6 ± 0.2 (99%); 44.0 ± 3.5 (1%) R^2 0.999	12.8 ± 0.2 (98%); 33.1 ± 1.2 (2%) R^2 0.999
**DCM**	3.1	14.1 ± 0.5 (93%); 31.9 ± 1.0 (7%) R^2 0.999	15.7 ± 0.5 (94%); 34.6 ± 1.7 (6%) R^2 0.999
**TOL**	2.4	11.8 ± 0.1 (97%); 39.2 ± 2.3 (3%) R^2 0.999	11.5 ± 0.1 (99%); 38.0 ± 1.9 (1%) R^2 0.999
**HEX**	0.1	7.6 ± 0.1 (99%); 40.8 ± 2.1 (1%) R^2 0.999	7.6 ± 0.1 (99%); 41.6 ± 2.5 (1%) R^2 0.998
**CHX**	0.0	10.4 ± 0.4 (98%); 37.8 ± 1.9 (2%) R^2 0.999	10.1 ± 0.1 (99%); 38.1 ± 1.8 (1%) R^2 0.999

Table S4A. Photophysical properties of **ANP** and **PANP**

	**ANP**								**PANP**							
**Solvents**	$^a\lambda_{abs}$ max	$^a\lambda_{em}$ max	$^b\varepsilon$	^{c}**Stokes**	^dE_S	ϕ_F	ϕ_{nrad}	^ek_F x10^7	$^a\lambda_{abs}$ max	$^a\lambda_{em}$ max	$^b\varepsilon$	^{c}**Stokes**	^dE_S	ϕ_F	ϕ_{nrad}	^ek_F x10^7
**DMF**	403	523	5590	5802	66.5	0.06	0.94	2.2	342, 356, 410	520	17070	5160	60.8	0.07	0.93	4.7
**ACN**	402	520	4501	5583	67.1	0.02	0.98	1.2	340, 354, 408	520	14921	5279	62.1	0.02	0.98	2.5
**THF**	396	508	6082	5567	64.0	0.15	0.85	8.4	340, 355, 393	506	18792	5682	62.8	0.13	0.87	4.5
**DCM**	402	504	4501	5034	61.2	0.52	0.48	6.6	343, 358, 387	498	18559	5759	63.6	0.34	0.66	5.6
**TOL**	395	503	3623	5436	63.4	1	0	15.7	343, 358, 388	494	16408	5530	63.8	0.24	0.76	1.7
**HEX**	374	469	2446	5416	66.5	1	0	13.6	324, 338, 353, 379	469	16163	5063	66.03	0.21	0.79	1.5

^{a}in nm; ^{b}in $M^{-1}cm^{-1}$; ^{c}in cm^{-1}; ^{d}in kcal mol^{-1}; ^{e}in s^{-1}

Table S4B. Values of the emission lifetimes of **ANP** and **PANP** in different solvents

**Solvents**	**Polarity**	**ANP (τ_1; τ_2) / ns**	**PANP (τ_1; τ_2) / ns**
**DMF**	6.4	2.70±0.12 (100%) R^2 0.947	2.13 ±0.09 (100%) R^2 0.986
**ACN**	5.8	1.67± 0.04 (100%) R^2 0.984	4.04 ± 0.11(100 %) R^2 0.982
**THF**	4.0	11.75± 2.19 (1 %); 1.78± 0.14 (99 %)	9.38± 0.76 (1 %); 2.21± 0.18 (99 %) R^2 0.995
**DCM**	3.1	7.85± 0.083 (100%) R^2 0.994	11.67± 1.76 (1 %); 1.77± 0.10 (99 %) R^2 0.996
**TOL**	2.4	6.34± 0.05 (100%) R^2 0.997	5.80± 0.04 (100%) R^2 0.998
**HEX**	0.1	7.35± 0.06 (100%) R^2 0.995	6.69± 0.08 (100%) R^2 0.995

Table S5. Photophysical properties of **ANA** and **PANA**

	ANA								PANA							
**Solvents**	[a]λ_{abs} max	[a]λ_{em} max	[b]ε	[c]**Stokes**	[d]E_S	ϕ_F	ϕ_{nrad}	[e]k_F x10^7	[a]λ_{abs} max	[a]λ_{em} max	[b]ε	[c]**Stokes**	[d]E_S	ϕ_F	ϕ_{nrad}	[e]k_F x10^7
**DMSO**	345, 359	n.a.	12334	n.a	n.a.	n.a	n.a	n.a	344, 358	n.a.	15558	n.a.	76.4	0.017	0.983	n.a
**DMF**	344, 357	396	18484	2489	77.5	0.028	0.972	n.a	343, 357	398	23455	2885	77.4	0.026	0.974	n.a
**ACN**	341, 355	396	9981	2646	78.3	0.055	0.945	n.a	341, 356	378, 393	20899	2645	78.1	0.034	0.966	n.a
**THF**	341, 354	390	17328	2697	78.4	0.038	0.962	n.a	341, 356	374, 390, 412	22959	3645	78.4	0.023	0.977	n.a
**DCM**	344, 359	376, 395	15940	2538	77.5	0.063	0.937	n.a	344, 358	378, 394, 418	24787	4009	77.5	0.026	0.974	n.a
**TOL**	343, 359	400	15801	2855	77.3	0.074	0.926	n.a	343, 359	400	21201	15801	77.3	0.05	0.95	n.a
**HEX**	325, 340, 355	n.a.	9416	n.a.	n.a.	n.a.	n.a.	n.a	323, 339, 354	n.a.	19318	n.a.	n.a.	n.a.	n.a.	n.a
**CHX**	325, 339, 355	n.a.	7625	n.a.	n.a.	n.a.	n.a.	n.a	324, 340, 355	n.a.	21115	n.a.	n.a.	n.a.	n.a.	n.a

[a]in nm; [b]in $M^{-1}cm^{-1}$; [c]in cm^{-1}; [d]in kcal mol^{-1}; [e]in s^{-1}

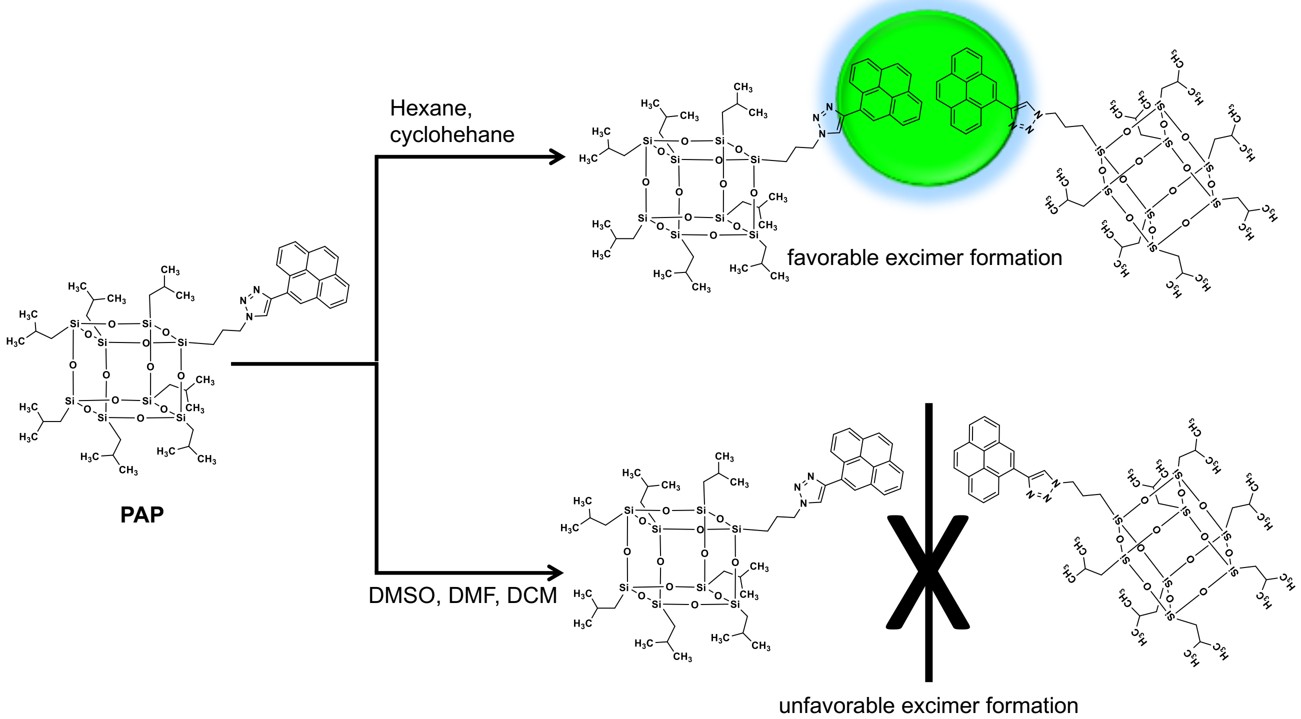

Figure S12. Proposed mechanism for the formation of excimer in **PAP**.

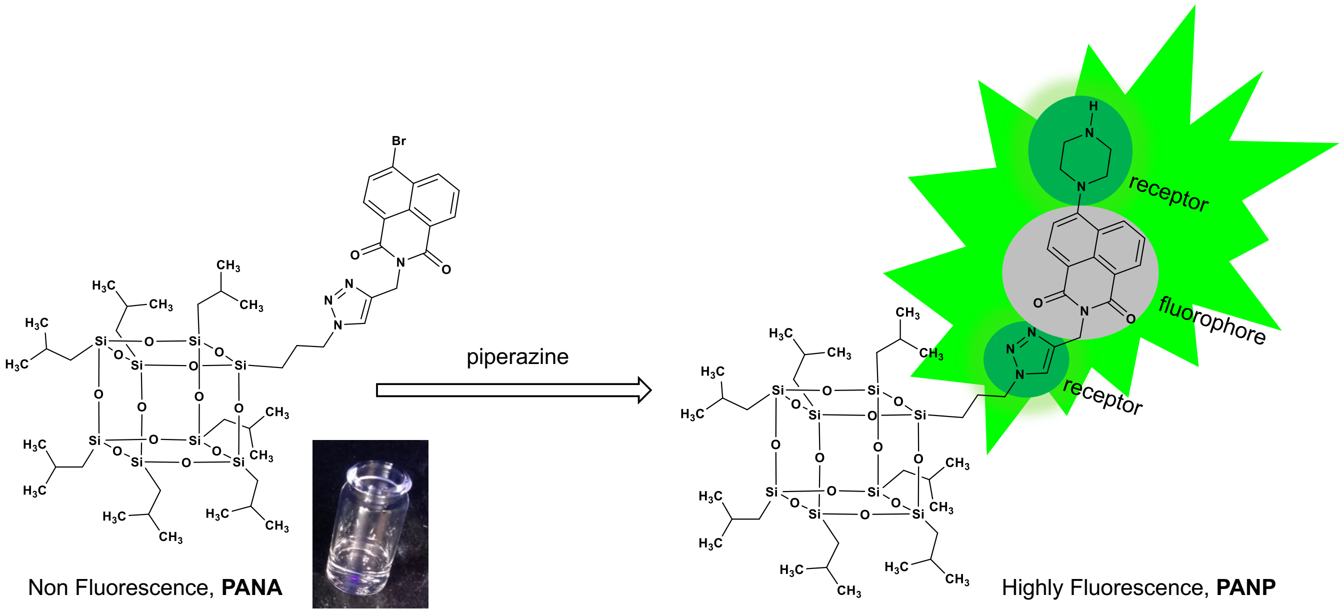

Figure S13. Design of **PANP** fulfilling “receptor1-fluorophore-receptor2” model.

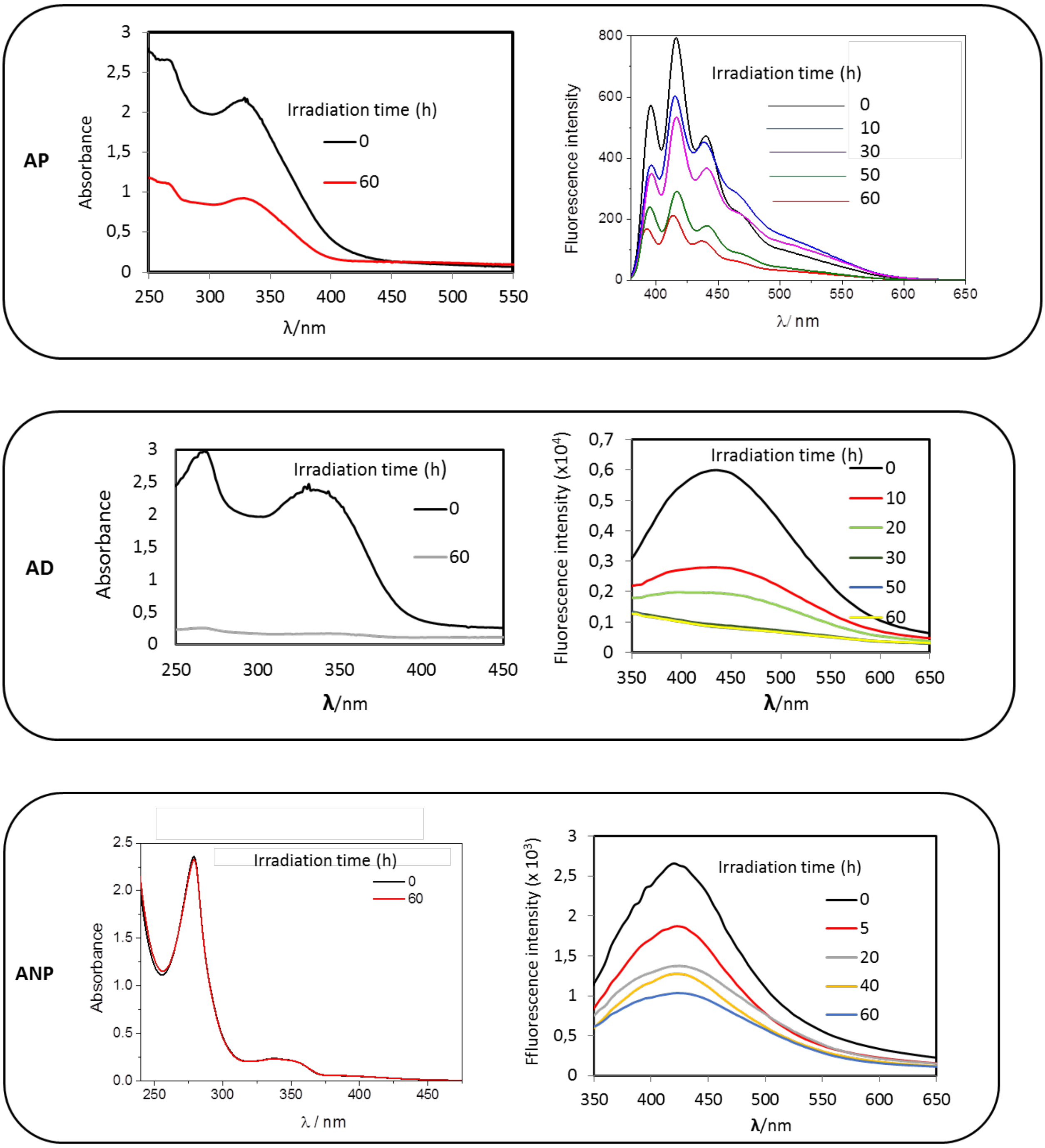

Figure S14. Absorption and emission profile of alkynyl dye substrate (**AP**, **AD** and **ANP**) after 60 h monochromatic irradiation for photostability studies.

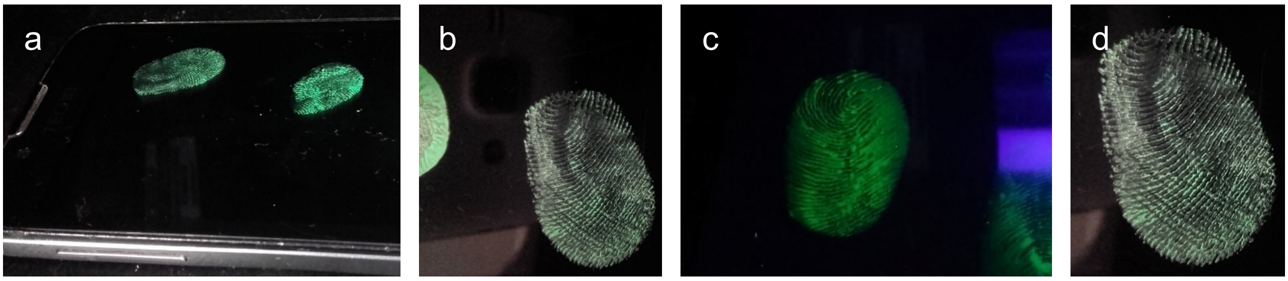

Figure S15. Images of fingerprints detection on phone (a, b) and glass surfaces (c, d).

Additional Supplementary Figures

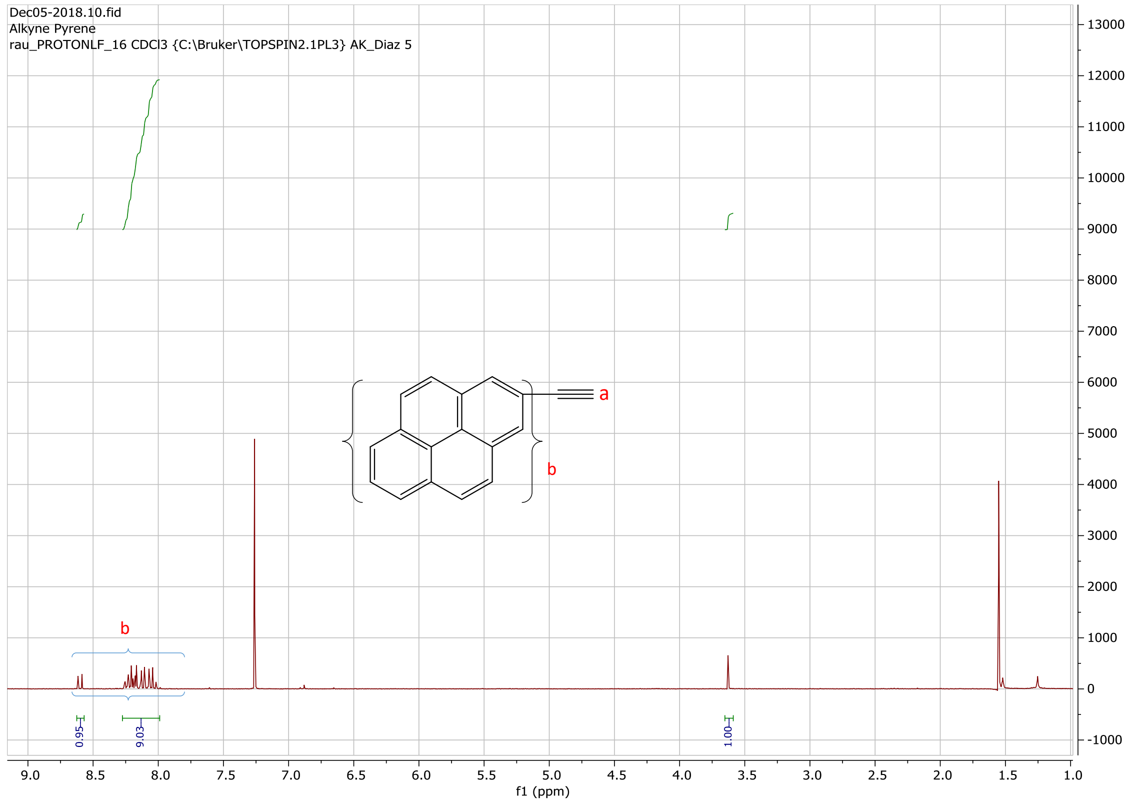

Figure S16. ^{1}H NMR spectrum of **AP**.

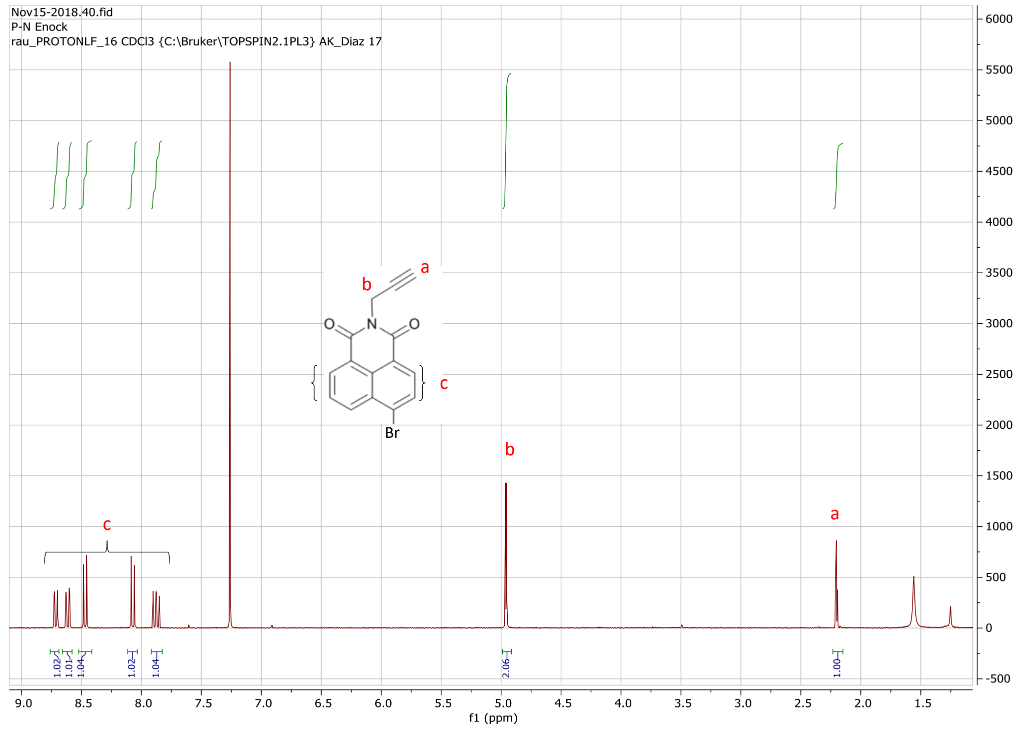

Figure S17. 1H NMR spectrum of **ANA**.

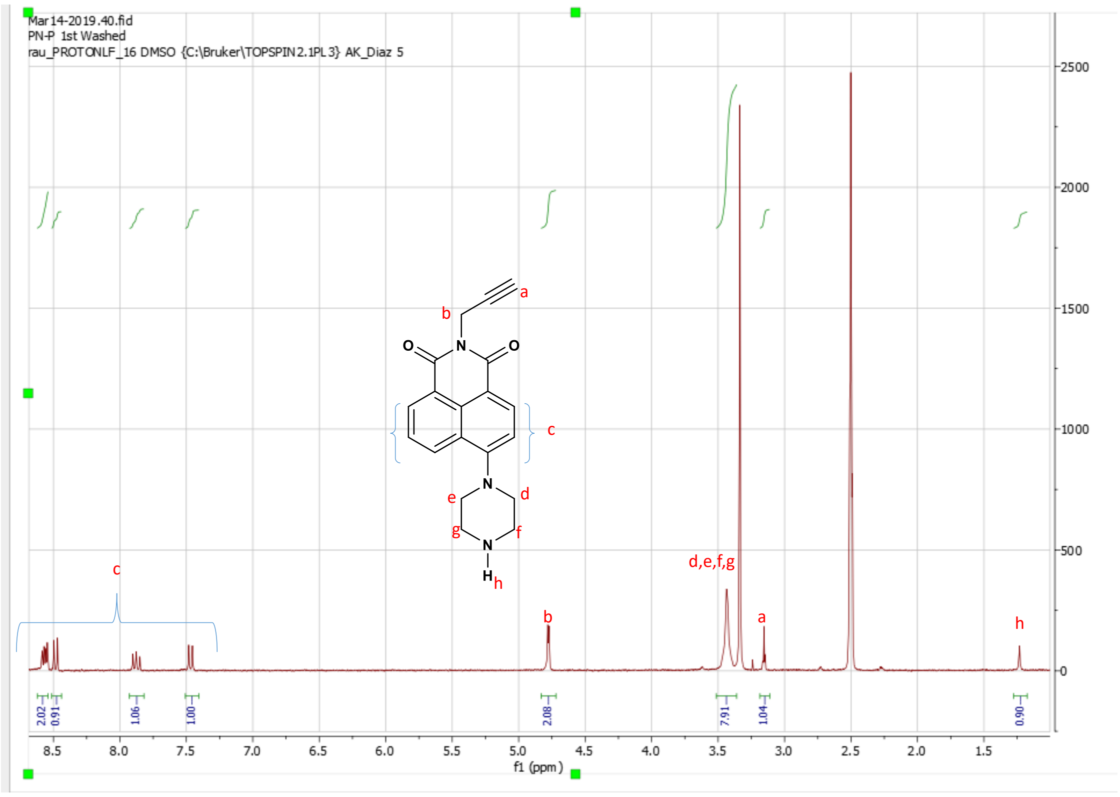

Figure S18. ^{1}H NMR spectrum of **ANP**.

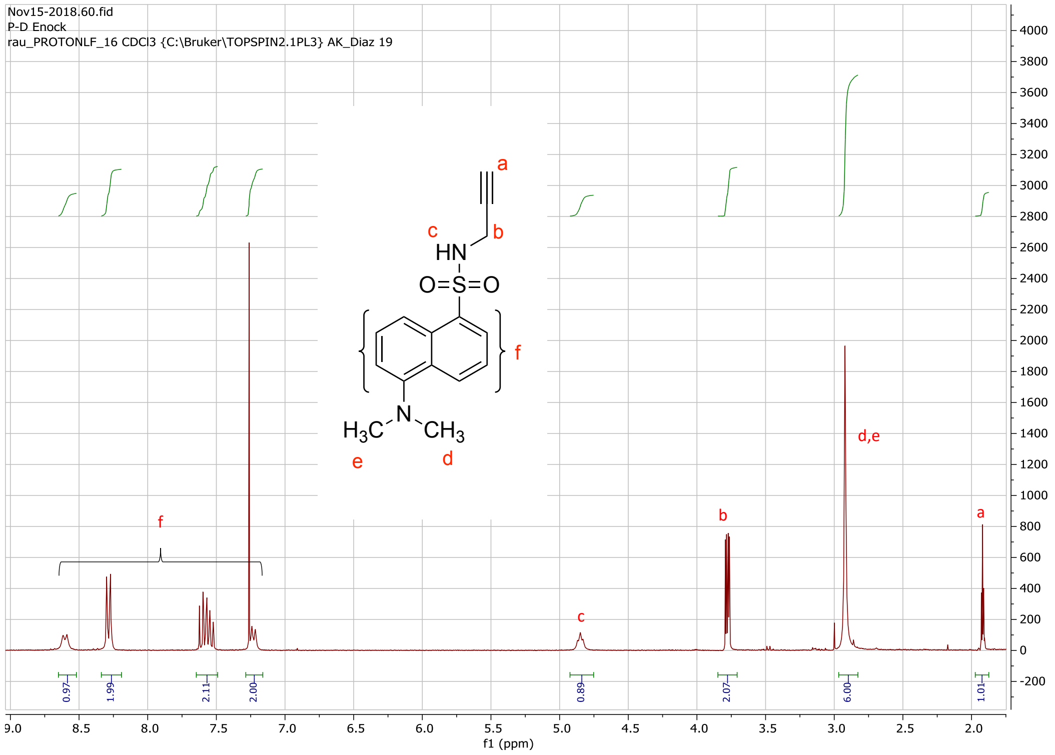

Figure S19. ^{1}H NMR spectrum of **AD**.

Author Contributions

E. O. Dare: experimental investigation, conceptualization, non-photophysical data acquisition, data interpretation, funding acquisition, writing of original draft. V.Vendrell-Criado: photophysical data acquisition, data interpretation. M. Consuelo Jiménez: supervision photophysical characterization, photophysical data discussion, funding acquisition, contribution to writing final draft. R. Pérez-Ruíz: supervision photophysical characterization, photophysical data discussion, funding acquisition, contribution to writing final draft. D. D. Díaz: scientific supervision, project and results discussion, funding acquisition, writing final draft.
